# Thrombospondin 1 enhances systemic inflammation and disease severity in acute-on-chronic liver failure

**DOI:** 10.1186/s12916-024-03318-x

**Published:** 2024-03-05

**Authors:** Hozeifa Mohamed Hassan, Xi Liang, Jiaojiao Xin, Yingyan Lu, Qun Cai, Dongyan Shi, Keke Ren, Jun Li, Qi Chen, Jiang Li, Peng Li, Beibei Guo, Hui Yang, Jinjin Luo, Heng Yao, Xingping Zhou, Wen Hu, Jing Jiang, Jun Li

**Affiliations:** 1 Precision Medicine Center, Taizhou Central Hospital (Taizhou University Hospital), Taizhou, 318000 China; 2 State Key Laboratory for Diagnosis and Treatment of Infectious Diseases, National Clinical Research Center for Infectious Diseases, National Medical Center for Infectious Diseases, Collaborative Innovation Center for Diagnosis and Treatment of Infectious Diseases, The First Affiliated Hospital, Zhejiang University School of Medicine, 79 Qingchun Rd., Hangzhou, 310003 China; 3 Key Laboratory of Cancer Prevention and Therapy Combining Traditional Chinese and Western Medicine, Tongde Hospital of Zhejiang Province, Hangzhou, China; 4 Department of Infectious Diseases and Liver Diseases, Ningbo Medical Center Lihuili Hospital, Affiliated Lihuili Hospital of Ningbo University, Ningbo, China; 5 Department of Pathology, The First Affiliated Hospital, Zhejiang University School of Medicine, Hangzhou, China; 6 Department of Infectious Disease, The First Affiliated Hospital of Anhui Medical University, Hefei, China

**Keywords:** Acute-on-chronic liver failure, Thrombospondin 1, Immune-metabolism disorder, Severity prediction, Inflammatory response, Hepatocellular apoptosis

## Abstract

**Background:**

The key role of thrombospondin 1 (THBS1) in the pathogenesis of acute-on-chronic liver failure (ACLF) is unclear. Here, we present a transcriptome approach to evaluate THBS1 as a potential biomarker in ACLF disease pathogenesis.

**Methods:**

Biobanked peripheral blood mononuclear cells (PBMCs) from 330 subjects with hepatitis B virus (HBV)-related etiologies, including HBV-ACLF, liver cirrhosis (LC), and chronic hepatitis B (CHB), and normal controls (NC) randomly selected from the Chinese Group on the Study of Severe Hepatitis B (COSSH) prospective multicenter cohort underwent transcriptome analyses (ACLF = 20; LC = 10; CHB = 10; NC = 15); the findings were externally validated in participants from COSSH cohort, an ACLF rat model and hepatocyte-specific THBS1 knockout mice.

**Results:**

*THBS1* was the top significantly differentially expressed gene in the PBMC transcriptome, with the most significant upregulation in ACLF, and quantitative polymerase chain reaction (ACLF = 110; LC = 60; CHB = 60; NC = 45) was used to verify that *THBS1* expression corresponded to ACLF disease severity outcome, including inflammation and hepatocellular apoptosis. THBS1 showed good predictive ability for ACLF short-term mortality, with an area under the receiver operating characteristic curve (AUROC) of 0.8438 and 0.7778 at 28 and 90 days, respectively. Enzyme-linked immunosorbent assay validation of the plasma THBS1 using an expanded COSSH cohort subjects (ACLF = 198; LC = 50; CHB = 50; NC = 50) showed significant correlation between THBS1 with ALT and γ-GT (*P* = 0.01), and offered a similarly good prognostication predictive ability (AUROC = 0.7445 and 0.7175) at 28 and 90 days, respectively. ACLF patients with high-risk short-term mortality were identified based on plasma THBS1 optimal cut-off value (< 28 µg/ml). External validation in ACLF rat serum and livers confirmed the functional association between THBS1, the immune response and hepatocellular apoptosis. Hepatocyte-specific THBS1 knockout improved mouse survival, significantly repressed major inflammatory cytokines, enhanced the expression of several anti-inflammatory mediators and impeded hepatocellular apoptosis.

**Conclusions:**

THBS1 might be an ACLF disease development-related biomarker, promoting inflammatory responses and hepatocellular apoptosis, that could provide clinicians with a new molecular target for improving diagnostic and therapeutic strategies.

**Graphical Abstract:**

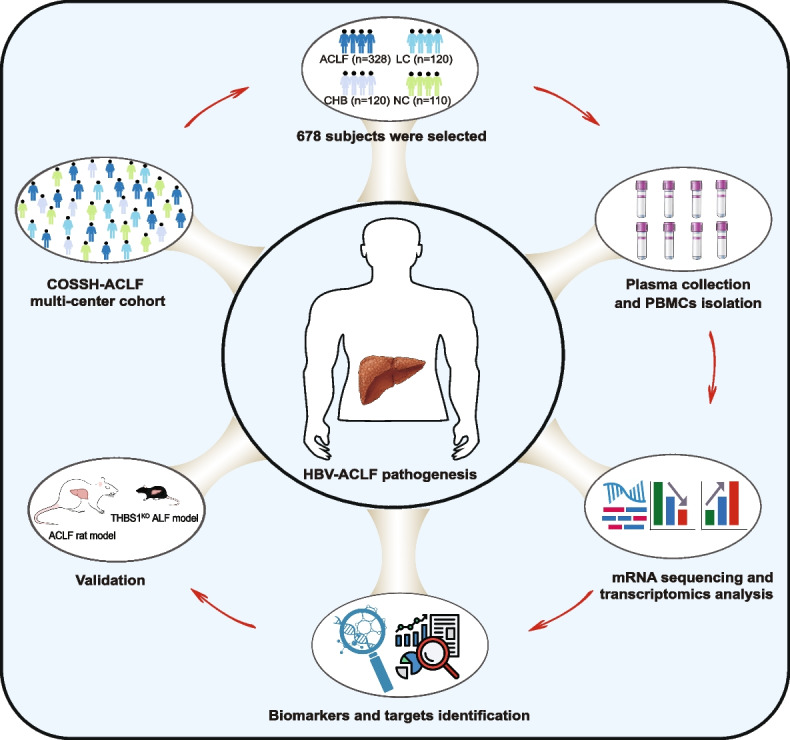

**Supplementary Information:**

The online version contains supplementary material available at 10.1186/s12916-024-03318-x.

## Background

Standing as distinct disease entity, acute-on-chronic liver failure (ACLF) develops from chronic liver disease subsequent to various insults and is characterized by severe disease, rapid development, poor prognosis and high mortality [1, 2]. ACLF depicted by regional phenotype specificity caused by different etiologies and precipitants. In Europe and the United States, the main etiology of ACLF is alcoholic cirrhosis, and the primary precipitants including bacterial infection, sepsis and systemic inflammation; while in China, the most common precipitant is hepatitis b virus (HBV), and HBV reactivation in infected patients represents the main trigger of ACLF [3].

Understanding the molecular mechanism of ACLF pathogenesis is essential for the development of effective diagnostic and therapeutic strategies. In this context, prominent dysregulation of immune and metabolic processes has been recognized as the key mechanism underlying ACLF pathogenesis, which was previously reported by the two major comprehensive studies worldwide, the Chronic Liver Failure (CLIF) Consortium Acute-On-Chronic Liver Failure in Cirrhosis (CANONIC) study and our recent large prospective multicenter Chinese Group on the Study of Severe Hepatitis B (COSSH) study on HBV-related ACLF (HBV-ACLF). Both studies highlighted either the intense systemic inflammatory response or an excessive immune response triggered by HBV exacerbation initiated immune-metabolic disorder, the key contribution factors that provokes multiorgan failure and worsen ACLF prognosis [4–7]. Furthermore, various studies have highlighted the potential roles of autoimmune hepatitis (AIH) and drug-induced liver injury (DILI) in the pathophysiology of ACLF. Typical AIH patients may develop ACLF following a second insult, possibly exacerbated by long-term immunosuppression [8]. In contrast, antitubercular agents and complementary and alternative medications are recognized as classical triggers of DILI leading to ACLF [9].

Recently, we emphasized the role of several potential biomarkers associated with the immune-metabolism dysregulation linked to HBV-ACLF prognosis, among which thrombospondin 1 (THBS1) was recognized within the top significantly differentially expressed key molecules [7]. THBS1, a member of the thrombospondin family, is a matricellular glycoprotein produced by various cells, including platelets, hepatocytes and other cells, where it can interact with multiple targets and receptors, such as CD36, integrins, CD47 and TGF-β, and participates in multiple functions, including inflammation, angiogenesis regulation, apoptosis and other cellular fate determinants [10, 11]. THBS1 is secreted in response to inflammation [12], and has been shown to be involved in tissue injury, inflammatory diseases, liver fibrosis and hepatic cancer [13], but there is no clear evidence regarding the involvement of THBS1 in ACLF pathogenesis and outcome.

**Fig. 1:**
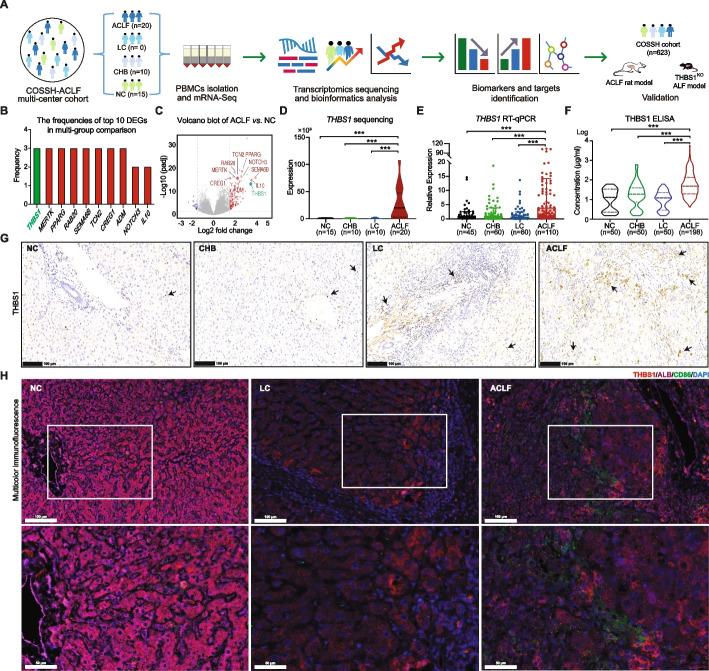
Identification of THBS1 as a potential key biomarker in HBV-ACLF pathogenesis. **A** Schematic representation of the study design and analysis strategy flow chart. To identify potential biomarkers, biobanked peripheral blood mononuclear cells (PBMCs) collected from patients in the Chinese Group on the Study of Severe Hepatitis B (COSSH) multicenter cohort underwent transcriptomic sequencing (ACLF = 20; LC = 10; CHB = 10; NC = 15), and the results were validated in humans, an ACLF preclinical rat model and THBS1^KO^ ALF mice. **B** Calculated frequencies of the top 10 genes (ranked by adjusted *P* value) that were identified in the multigroup comparison. **C** The top 10 genes are shown in the volcano plot of the ACLF *vs.* NC groups. The vertical dashed lines indicate the threshold for the fourfold difference in abundance. The horizontal dashed line indicates the adjusted *P* value = 0.05 threshold. Red represents significantly upregulated genes (*THBS1* in green), while blue represents significantly downregulated genes. The node size represents the frequency of key genes. **D**-**F ***THBS1* expression profile in the sequencing group (**D**), and RT–qPCR validation of *THBS1* expression (**E**) in human PBMCs (ACLF = 110; LC = 60; CHB = 60; NC = 45). *** *P* value < 0.001, *GAPDH* was set as a reference control gene. Serum levels of THBS1 measured by ELISA (**F**) and analyzed using the Mann–Whitney U test (ACLF = 198; LC = 50; CHB = 50; NC = 50). *** *P* value < 0.001 *vs.* NC group. **G** Representative immunohistochemical staining of THBS1 in liver tissues (black arrow) from patients in the HBV-ACLF, LC, CHB and NC groups. **H** Quantitative multiplex tyramide signal amplification (TSA) immunofluorescent images showing THBS1 expression intensities in liver tissues from patients in the HBV-ACLF, LC and NC groups, stained with THBS1 (red), a hepatocyte marker (ALB, purple) and a macrophage marker (CD86, green) and a nuclear marker (DAPI, blue)

On this background, we hypothesized that THBS1 expression is positively correlated with the typical disease features of ACLF, namely, inflammation and hepatocellular apoptosis. Consequently, in this work, we first analyzed the expression profile of THBS1 as a diagnostic/prognostic biomarker, predictor of ACLF severity and disease outcome in a total of 568 subjects with HBV-related etiologies and 110 healthy normal controls, as well as in our previously established ACLF rat model. Notably, we developed a hepatocyte-specific THBS1 knockout (THBS1^KO^) mouse model to validate the hepatoprotective capabilities following intoxication, and the overall study design is summarized in Fig. 1A.

## Methods

### Study design, participant recruitment and clinical data collection

The biobanked peripheral blood mononuclear cells (PBMCs) collected from patients in the COSSH multicenter cohort underwent transcriptomic sequencing. PBMCs were used since they are sensitive bystanders in peripheral blood because they are easier to obtain than liver tissues and their results are more easily translated [14]. Briefly, PBMCs isolated from 330 patients representing the spectrum of HBV-related liver diseases, including chronic hepatitis B (CHB, *n* = 70), liver cirrhosis (LC, *n* = 70) and ACLF (*n* = 130), and healthy normal control volunteers (NC, *n* = 60) were subjected to functional synergy analysis and transcriptomic phenotyping (ACLF = 20; LC = 10; CHB = 10; NC = 15) to reveal the potential biomarkers associated with HBV-ACLF pathogenesis. The general clinical characteristics of the participants are elaborated in Additional file 1: Table S1. For enzyme-linked immunosorbent assay (ELISA) external validation, we used the plasma of an expanded participants (ACLF = 198; LC = 50; CHB = 50; NC = 50) independently recruited from the COSSH-ACLF multicenter cohort. To ensure data quality and minimize selection bias, all ACLF patients were randomly selected using stratified random sampling based on the prevalence of ACLF grade in the COSSH cohort (ACLF‐1, 62.6%; ACLF‐2, 28.3%; ACLF‐3, 9.1%). The general clinical characteristics of the ACLF subjects participated in ELISA validation are elaborated in Additional file 1: Table S2.

### Subject definitions and diagnostic criteria for ACLF, LC and CHB

Diagnosis of ACLF was based on COSSH-ACLF criteria as previously described [15]. Defined by these criteria, HBV-ACLF represents a complicated syndrome with high short-term mortality that develops in patients with HBV-related chronic liver disease, regardless of the presence of cirrhosis, and it is marked by the acute deterioration of liver function and hepatic and/or extrahepatic organ failure. HBV-ACLF is categorized into three grades (ACLF-1, ACLF-2 and ACLF-3) based on the extent of organ failure(s). LC was defined as patients with stable compensated cirrhosis, which was diagnosed on the basis of previous liver biopsy results, clinical evidence, laboratory tests, and endoscopic (esophageal and gastric varices) and radiological imaging of portal hypertension and/or liver nodularity, as previously described [7]. The enrolment criteria for the patients with CHB corresponded to the 2016 American Association for the Study of Liver Diseases (AASLD) guidelines [16], and normal healthy volunteers presented with normal physical examination. We excluded patients according to the following criteria: 1) younger than 18 years or older than 80 years; 2) with evidence of pregnancy; 3) concurrent hepatocellular carcinoma or other tumors; 4) severe extrahepatic diseases; 5) debilitating mental disease; 6) patients receiving immunosuppressive drugs for other reasons; and 7) inability to comply with the study protocol [15, 17]. Patients were receiving antiviral therapy to control their viral loads, necessary measures for integrative treatment, including treatment administration for ascites, HE, and bacterial infections, and renal replacement for hepatorenal syndrome. All participants enrolled in our study were liver transplantation-free, as liver transplantation can significantly impact survival analysis, and the detailed subject definition and characterization are presented in the Additional file 2: Methods.

### Preparation for mRNA sequencing and gene expression analysis

For mRNA-seq, PBMCs were isolated from total blood using Ficoll-Paque™ PLUS medium (GE Healthcare, Uppsala, Sweden) from patients and volunteers. Total RNA was extracted from PBMCs using TRIzol reagent (Ambion, Carlsbad, CA). Sequencing libraries were prepared according to the manufacturer’s instructions (TruSeq® RNA LT Sample Prep Kit v2, Illumina, San Diego, CA), including purifying and fragmenting mRNA, synthesizing first-strand cDNAs, synthesizing second-strand cDNAs, performing end repair by adenylating the 3’ ends, ligating adapters, and enriching DNA fragments. The pooled library consisted of sequences with lengths of approximately 250 nucleotides. The library was sequenced using the HiSeq 2500 sequencing platform (Illumina, USA) as highlighted in the Additional file 2: Methods [18–21].

### Animal models

#### ACLF rat model

A stable ACLF rat model that entirely mimics the clinical pathogenesis of the disease has been established on the basis of LC, as reported elsewhere [22]. Briefly, male Sprague–Dawley rats of specific pathogen-free grade and weighing 60–80 g (age 4 weeks old) were randomly allocated into two groups (control group: *n* = 10; ACLF group: *n* = 30). In the ACLF group, rats were intraperitoneally administered porcine serum at 2 mL/kg twice a week for 12 consecutive weeks to generate LC. Next, rats with stable LC were intraperitoneally injected with a combination of D-galactosamine (D-gal, 800 mg/kg) and bacterial lipopolysaccharide (LPS, 100 μg/kg) to induce acute liver failure based on chronic LC. Meanwhile, rats in the control group were administered only normal saline. Animals were sacrificed at 0 and 72 h following acute insult, and serum and liver tissues were collected from rats at different ACLF disease stages as well as from NC rats and used to confirm the significant involvement of THBS1 in ACLF pathogenesis via transcriptome, histology and molecular biology analyses.

#### Acute liver failure mouse model

To generate an acute liver failure (ALF) mouse model, the hepatocyte-specific THBS1^KO^ mice (20–22 g body weight, 6–8 weeks old, C57BL/6 background, *n* = 50) and their age/weight-matched wild-type littermates (WT, *n* = 50) were obtained from Cyagen Biosciences (Guangzhou, China), and the details on the source strain, knockout technique and offspring bred are elaborated in the Additional file 2: Methods. Both hepatocyte-specific THBS1^KO^ and WT mice were maintained in a specific pathogen-free facility at the Zhejiang Academy of Medical Sciences animal center (animal license number SCXK 2019-0002, Hangzhou, China) and then subclassified into a control group that received vehicle and an ALF model group that received acute intraperitoneal injections of D-gal (600 mg/kg) and LPS (50 µg/kg) to induce liver failure. The combination of D-gal and LPS has been well recognized pharmacological approach for ALF induction by single intraperitoneal injection, as previously described [23, 24]. Other than those used for survival analysis, mice were sacrificed at designated time-points, and blood and liver tissues were collected for further analysis.

### Real-time quantitative polymerase chain reaction (RT-qPCR)

RT–qPCR validation was carried out with PBMCs from COSSH cohort subjects (ACLF = 110; LC = 60; CHB = 60; NC = 45) to confirm the results of the transcriptomic analysis. For animal models, snap-frozen liver tissues were lysed and total RNA was extracted and reversed transcribed, then PCR was performed using a two-step protocol with specific primers, TB Green DYE II (Takara, Beijing, China), and an ABI 7500HT instrument (Thermo Fisher, Waltham, MA) according to the manufacturer’s instructions. The amount of cDNA was optimized to ensure that the amplification of both control genes and cDNAs of interest occurred in the exponential phase. Transcript values, expressed as the relative mRNA level of specific target genes, were normalized against glyceraldehyde-3-phosphate dehydrogenase (GAPDH) mRNA levels, which served as a reference control, using the comparative Ct method [25]. The specific primers used in this study (Additional file 1: Table S3) were synthesized, purified and quality-inspected by Sangon Biotech (Shanghai, China).

### ELISA and cytokine analysis

Plasma levels of THBS1 were quantified using ELISA kit on samples from COSSH cohort subjects (ACLF = 198; LC = 50; CHB = 50; NC = 50) according to the manufacturer instruction (Abcam, UK). For ACLF rats, validation of THBS1 expression was carried out using commercially available kit (Abbexa, USA). The expression profiles of cytokine panels in rat serum were measured using antibody-multiplexed sandwich ELISA-based quantitative array following assay guidelines (RayBiotech, China), and data analyzed using manufacturer’s software.

### Cut-off determination and risk assessment

For the convenience of potential clinical application, we identified the optimal cut-off value of the plasma THBS1 expression level in relation to the ACLF mortality. Consequently, this cut­off value was used to further stratify patients into low- or high-risk death groups, employing the X-tile program (Version 3.6.1, Yale University School of Medicine) as previously described [26]. The cumulative survival analysis was performed using the Kaplan‒Meier method, and the log-rank test was used to assess the significance of differences between the low-risk and high-risk groups.

### Histopathology and immunohistochemistry

Pathological, immunofluorescent and immunohistochemical staining was performed on hepatic tissues with the corresponding stains and antibodies using a Leica Bond-Max staining according to the manufacturer’s instructions (Leica Biosystems Inc., Melbourne, Australia). For THBS1 localization, an Opal™ multicolor immunofluorescence tyramide signal amplification (TSA) technology was performed on formalin-fixed, wax-embedded liver tissue samples from COSSH cohort, and then slides were photographed by either NanoZoomer 2.0RS Digital slide scanner (Hamamatsu, Japan) or Caseviewer (3DHISTECH Ltd., Hungary).

### Western immunoblotting

A total of 70 mg of liver tissue was homogenized in radioimmunoprecipitation assay buffer with pre-added phosphatase and protease inhibitors (Beyotime, China), and the total protein concentrations were determined using a Pierce^®^ Protein Assay kit (Thermo, United States). SDS–PAGE gels (10–20% v/v) were loaded with 40–60 µg of protein per tissue sample diluted in NuPAGE® LDS sample buffer (Thermo Fisher, United States) and transferred to polyvinylidene difluoride membranes (Millipore, USA). Following nonspecific blocking with QuickBlock™ (Beyotime, China), the membranes were incubated with different primary and secondary antibodies. Finally, the expression of each target protein was digitally detected by ChemiScope (Clinx, China). The antibodies used in this study are listed in Additional file 1: Table S4. All experiments were performed in triplicate, and the protein expression was assessed relative to that of β-actin, which was used as an internal control. The whole uncropped images of the original western blots are provided as Additional file 3: Data File.

### Statistical analysis

Unless indicated otherwise, the experimental data, presented as the mean ± SD, were statistically analyzed using Prism 8 (GraphPad Software, Inc., CA, USA) or R/RStudio software (http://www.R-project.org, version 4.0.3). Significance was determined using analysis of variance (ANOVA), and Student’s *t* test was applied for two-group comparisons. *P* values ≤ 0.05 were considered indicative of significance. The area under the receiver operating characteristic curve (AUROC) was calculated to assess the accuracy of THBS1 in predicting short-term mortality in ACLF. Pearson’s correlation coefficient (r) was used to assess the correlation of THBS1 compared to that of clinical biomarkers and different prognostic scoring systems, including the Chinese Group on the Study of Severe Hepatitis B-ACLF II score (COSSH-ACLF IIs), COSSH-ACLF score (COOSH-ACLFs), Chronic Liver Failure-Consortium ACLF score (CLIF-C ACLFs), Model for End-stage Liver Disease score (MELDs), MELD-sodium score (MELD-Nas), and Child-Turcotte-Pugh score (CTP), and a correlation plot was generated using the R packages “ggplot2” and “corrplot”.

## Results

### Transcriptome analysis revealed THBS1 as a key biomarker correlated with ACLF pathogenesis

To identify the key molecular biomarkers related to ACLF pathophysiology, disease severity and outcome, multiple pairwise comparisons of gene expression from PBMCs were performed (starting from NC to CHB to LC to ACLF). The frequency count data from the multigroup pairwise comparison among the different disease stages identified *THBS1* as the top significantly differentially expressed gene (DEG) in the PBMC transcriptome (Fig. 1B), indicating that it was significantly differentially expressed in most comparisons among the four groups. Furthermore, Fig. 1C displays the DEGs between the ACLF *vs.* NC groups, among which *THBS1* was the most significantly upregulated gene, highlighting the potential of this biomarker candidate to predict the ACLF clinical course and/or outcome. Importantly, the *THBS1* profile showed specific overexpression in the ACLF status relative to CHB/LC in the sequencing analysis (Fig. 1D), whereas RT–qPCR and ELISA validations performed on PBMCs and serum derived from different groups in the COSSH cohort, respectively, further verified the consistency of the THBS1 upregulation profile in ACLF disease status (Fig. 1E, F).

Moreover, our results showed different expression patterns of *THBS1* in ACLF patients; hence, we further subdivided the ACLF group into low- and high-THBS1 groups based on the median value of the relative *THBS1* expression. Patients with high THBS1 expression (*n* = 49) showed elevated levels of ALT, AST, Cr, platelet count, INR, as well as multi-organ failure and a poor prognosis for transplant-free short-term mortality (Additional file 1: Table S5).

THBS1 protein expression was also detected by immunohistochemistry (IHC) in liver tissues derived from NC, CHB, LC and ACLF patients in the COSSH cohort tissue bank (Fig. 1G), which showed progressively higher expression from CHB to LC to ACLF. To overcome the challenge of the co-localization of cellular sources that express THBS1, we applied multiplex TSA technology [27], to visualize multiple cell markers (Additional file 2: Methods). Interestingly, the expression intensities of THBS1, a hepatocyte marker (albumin, ALB) and a hepatic Kupffer cell marker (CD86) detected by multicolor immunofluorescence signal amplification revealed that THBS1 was observed together with either ALB or CD86 markers (Fig. 1H), indicating the THBS1 expression potential of hepatocytes and CD86^+^-Kupffer cells following ACLF onset. This could verify THBS1 expression in various cell types, including hepatocytes, immune cells and PBMCs.

### The potential of THBS1 as a predictor of HBV-ACLF disease severity and outcome

To characterize the THBS1 expression profile in the ACLF disease severity and outcome, we sub-classified the ACLF group based on the overall outcome (90-day short term mortality). As outlined in Table 1, 47 (46.0%) patients in the ACLF group died, and the remaining 55 (54.0%) survived the 90-day follow-up phase. As anticipated, significant hepatic dysfunction, manifested as an elevated TB level (*P* < 0.001) and INR (*P* < 0.05), as well as extrahepatic organ failure, was predominant in the ACLF deceased group. Except for the CTP, the calculated severity scores (COSSH-ACLF IIs, COSSH-ACLFs, CLIF-C ACLFs, MELD-Nas and MELDs) of the ACLF deceased group were significantly higher (*P* < 0.001) than those of the ACLF survival group.

**Table 1:** Clinical characteristics of ACLF patients with different outcomes at 90 days^a^

**Characteristics**^**b**^	**ACLF-S** ***n* = 55**	**ACLF-D** ***n* = 47**
**Age (yrs.)**	44.7 ± 11.0	46.6 ± 12.6
**Male (No.)**	87.3% (48)	85.1% (40)
**HBV-DNA level (IU/ml)**		
2 × 10^2^–2 × 10^4^	27.3% (15)	25.6% (12)
2 × 10^4^–2 × 10^6^	52.7% (29)	36.2% (17)
> 2 × 10^6^	20.0% (11)	38.3% (18)
**Laboratory data**		
Alanine aminotransferase (U/L)	232.0 [90.0, 550.0]	368.0 [104.0, 874.0]
Aspartate aminotransferase (U/L)	140.0 [90.50, 194.0]	146.0 [105.0, 438.0]
Albumin (g/dL)	31.4 ± 4.1	30.6 ± 5.0
Total bilirubin (µmol/l)	329.7 ± 107.9	416.2 ± 130.9***
Alkaline phosphatase (U/L)	131.0 [95.0, 162.0]	139.0 [120.0, 173.0]
γ-Glutamyl transpeptidase (U/L)	77.50 [59.0, 118.4]	81.0 [52.0, 125.0]
Creatinine (μmol/L)	66.0 [54.5, 72.0]	69.0 [59.5, 100.5]*
Sodium (mmol/L)	137.0 [135.0, 139.0]	137.0 [134.5, 139.0]
White blood cell count (10^9^/L)	7.3 [5.9, 9.3]	8.3 [6.6, 11.8]
Hemoglobin (g/L)	125.3 ± 18.0	124.5 ± 22.5
Hematocrit (%)	35.6 ± 5.4	35.5 ± 6.1
Platelet count	132.0 [85.0, 163.5]	104.0 [70.5, 129.5]*
INR	1.9 [1.7, 2.2]	2.50 [2.3, 3.3]***
Alpha fetoprotein	179.8 [66.9, 266.8]	66.3 [29.3, 140.4]
**Organ failure (No.)**		
Liver	96.4% (53)	97.9% (46)
Coagulation	18.2% (10)	55.3% (26)***
Kidneys	1.8% (1)	12.8% (6)
Brain	1.8% (1)	6.4% (3)
Lungs	0	0
Circulation	0	0
**ACLF grade**		Significance
ACLF-1	80.0% (44)	36.2% (17)
ACLF-2	20.0% (11)	55.3% (26)
ACLF-3	0	8.5% (4)
**Severity score**		
COSSH-ACLF IIs	6.7 [6.4, 7.3]	7.6 [7.3, 8.1]***
COSSH-ACLFs	5.6 [5.3, 6.1]	6.7 [6.2, 7.0]***
CLIF-C ACLFs	39.0 ± 5.6	44.5 ± 6.2***
MELD	22.1 ± 4.0	28.6 ± 7.1***
MELD-Na	23.6 [20.1, 26.1]	29.0 [25.1, 32.7]***
CTP	9.0 [9.0, 10.0]	10.0 [9.0, 11.0]

Data are expressed as the mean ± standard deviation (SD), median (p25, p75) or percentage (number of patients)

*ACLF-S* ACLF survival group, *ACLF-D* ACLF deceased group, *COSSH-ACLF IIs* COSSH-ACLF II score, *COSSH-ACLFs* COSSH-ACLF score, *CLIF-C ACLFs* CLIF Consortium ACLF score, *MELDs* Model for end-stage liver disease score, *MELD-Nas* MELD-sodium score, *CTP* Child-Turcotte-Pugh, *THBS1* Thrombospondin 1

^*^P < 0.05, **P < 0.01 and ***P < 0.001 for comparisons between the groups

^a^Twelve patients with ACLF underwent liver transplantation and were considered lost to follow-up in the mortality rate calculation. Six patients with ACLF were lost to the 28-day follow-up analysis, and 16 patients were lost to the 90-day follow-up analysis

^b^The number of ACLF patients were derived from both the sequencing and RT-qPCR groups

**Fig. 2:**
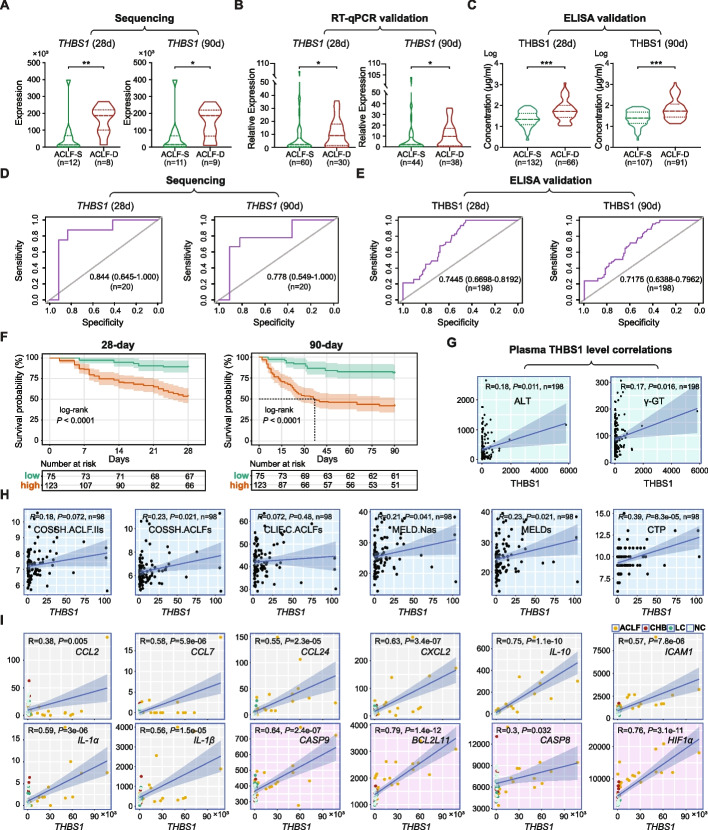
Influences of THBS1 on HBV-ACLF prognosis and short-term mortality. **A** Human PBMC transcriptome expression profile of *THBS1* in HBV-ACLF deceased group (*n* = 8/9) was significantly higher than HBV-ACLF survival group (*n* = 12/11) at 28/90 days. **B** PCR validation in human PBMCs confirmed the significant difference between HBV-ACLF deceased group (*n* = 30/38) and HBV-ACLF survival group (*n* = 60/44) at 28/90 days, * *P* < 0.05, ** *P* < 0.01. Twelve patients with ACLF underwent liver transplantation and were considered lost to follow-up in the mortality rate calculation. Six patients with ACLF were lost to the 28-day follow-up analysis, and 16 patients were lost to the 90-day follow-up analysis. **C** Plasma THBS1, measured by ELISA, verified the significant difference between HBV-ACLF deceased group (*n* = 66/91) and HBV-ACLF survival group (*n* = 132/107) at 28/90 days, *** *P* < 0.001. **D**, **E** Accuracy of THBS1 in predicting ACLF short-term mortality was evaluated through the area under the receiver operating characteristic curve (AUROC) in the sequencing group (**D**), and plasma THBS1 in the validation group (**E**) for 28 and 90 days. **F** Kaplan‒Meier curves of survival probability stratified by plasma THBS1 level at 28 days (left) and 90 days (right). Level of significance: *P* < 0.0001 (log-rank test). The number of patients at risk is shown for each observation period. **G** The correlation between serum THBS1 and the clinical parameters ALT and γ-GT (*n* = 198). **H** Correlations between *THBS1* expression in PBMCs and the different scoring systems (*n* = 98); *THBS1* was significantly (*P* < 0.05) positively correlated with COSSH-ACLFs, MELD-Nas, MELDs and CTP, whereas no significant (*P* > 0.05) correlation existed with either COSSH-ACLF IIs or CLIF-C ACLFs. **I** The correlation between *THBS1* levels and selected inflammatory mediators (grey) and apoptotic targets (pink) in the human PBMC transcriptome in the four groups comparison (ACLF *vs.* LC *vs.* CHB *vs.* NC). Pearson’s correlation coefficient (r) was used for the assessment of the correlations, and a correlation plot was generated using the R corrplot package

Next, the potential of THBS1 as a predictor of ACLF severity and outcome was evaluated, and our results showed that *THBS1* expression profile in the PBMCs from the ACLF deceased group was significantly higher than that from the ACLF survival group at 28 and 90 days (*P* < 0.01 and *P* < 0.05, respectively), in the sequencing analysis as demonstrated in Fig. 2A, whereas PCR and ELISA validations confirmed the significance of the THBS1 expression in the ACLF deceased group (*P* < 0.05 and *P* < 0.001, respectively) at 28 and 90 days (Fig. 2B, C). To support these findings, the accuracy of THBS1 expression in predicting ACLF short-term mortality was evaluated by AUROC measurement, in which the calculated AUROC for the sequencing group showed good prediction capabilities of 0.8438 and 0.7778 at 28 and 90 days, respectively, as illustrated in Fig. 2D. The AUROC analysis of the plasma THBS1 in the validation group offered a similarly good THBS1 prognostication predictive ability with an AUROC of 0.7445 and 0.7175 at 28 and 90 days, respectively (Fig. 2E).

### Risk stratification analysis and clinical correlations of THBS1

For potential clinical translation, the optimal cut-off of plasma THBS1 determined using X-tile software was 28 µg/ml. Subsequently, ACLF subjects were allocated into two groups based on the optimal cut-off value of the plasma THBS1 level: low-risk group (plasma THBS1 level < 28 µg/ml, *n* = 75) and high-risk group (plasma THBS1 level ≥ 28 µg/ml, *n* = 123). Patients in the high-risk group showed elevated levels of ALT, AST, γGT, platelet count, as well as multi-organ failure and a poor prognosis for transplant-free short-term mortality, and the overall clinical characteristics of patients in the low- and high-risk groups are detailed in Additional file 1: Table S6. Interestingly, survival analysis with Kaplan‒Meier performed using the log-rank test showed a significant difference (*P* < 0.0001) between the low- and high-risk groups at 28- and 90-day mortality (Fig. 2F).

Importantly, correlation analysis of plasma THBS1 and liver function-related biomarkers, ALT and γ-GT, exhibited significant positive correlations (*P* < 0.05), as indicated in Fig. 2G. Furthermore, the *THBS1* was significantly correlated with the COSSH-ACLFs (*P* < 0.05), MELD-Nas (*P* < 0.05), MELDs (*P* < 0.05), and CTP (*P* < 0.001). However, no significant correlation existed between *THBS1* and COSSH-ACLF IIs or CLIF-C ACLFs (Fig. 2H). Pearson’s correlation analysis of the PBMC transcriptomic data showed significant positive correlations between *THBS1* and randomly selected inflammatory-related cytokine genes, such as *CCL2*, *CCL7*, *CCL24*, *CXCL2*, *ICAM1*, *IL-1α*, *IL-1β*, and *IL-10*, and apoptosis-related genes, such as *Bcl2*, *Caspase-8*, *HIF1α* and *Caspase-9* (Fig. 2I). The detailed *THBS1* correlations with inflammatory and apoptotic markers in the PBMC transcriptomes of the different groups are provided in Additional file 4: Fig. S1. Overall, THBS1 might be considered a new molecular biomarker for predicting the ACLF disease severity and short-term outcome, whereas a possible combination of THBS1 with the existing prediction models could positively improve the accuracy of ACLF prognostication.

### Evidence of THBS1 overexpression in the ACLF rat

**Fig. 3:**
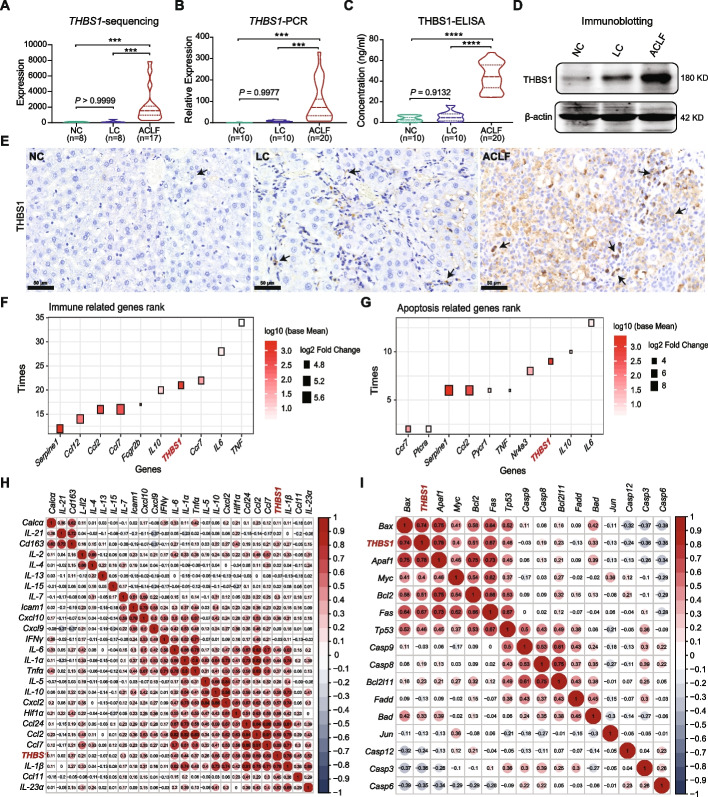
Expression profile of THBS1 in the ACLF preclinical rat model. The relative expression levels of *Thbs1* were measured by **A** sequencing analysis and validated by **B** RT–qPCR analysis using liver tissues from rats at different disease stages (ACLF = 17; LC = 8; NC = 8), *** *P* < 0.001, *Gapdh* was used as a reference control gene. **C** THBS1 levels were measured by ELISA in the serum from rats at different disease stages (ACLF = 20; LC = 10; NC = 10), **** *P* < 0.0001. **D** Representative immunoblotting data showing the THBS1 protein expression profile in rat liver tissues at different disease stages; β-actin was used as a loading control. **E** Representative immunohistochemical staining of THBS1 (black arrow) in liver tissues collected from rats at different disease stages. **F**, **G** The frequencies of the top genes appearing in **F** immune-related and **G** apoptosis-related biological processes. The color intensity represents the mean expression of genes in ACLF rat hepatic tissues. The size of the squares represents the log2(fold change) of the comparison between ACLF and LC groups. **H**, **I** Serum THBS1 was positively correlated with the top key genes related to **H** immune microenvironment and **I** apoptotic markers in ACLF rat hepatic tissues

To validate the role of THBS1 as a potential biomarker of ACLF, we analyzed the expression patterns of THBS1 in our previously established ACLF rat model. Liver tissues and serum from rats at different ACLF disease stages were collected and subjected to gene transcriptome, qPCR, ELISA and IHC analysis. The *THBS1* expression profile in the liver tissue of ACLF rats in the sequencing analysis (Fig. 3A) showed significant elevation during the progression from NC/LC to ACLF (*P* < 0.001), which was consistent with the gene expression profile previously assessed in human PBMCs from COSSH cohort. Verification by RT-qPCR analysis using liver tissues (Fig. 3B) showed significantly higher *Thbs1* expression (*P* < 0.001) in the ACLF group in comparison to the NC/LC groups. As illustrated in Fig. 3C, in accordance with the manufacturer’s guidelines (Abbexa, USA), ELISA measurement revealed a significant increase in serum THBS1 levels in the ACLF rats (*P* < 0.0001) regardless of those in the NC and/or LC groups. These outcomes were further validated by immunoblotting data (Fig. 3D) and immunohistochemical analysis (Fig. 3E) using ACLF rat liver tissues, which demonstrated a significant elevation in hepatic THBS1 protein expression during ACLF progression compared to the reduced expression intensities in both the NC and LC groups. Taken together, irrespective of the precipitating event, the above results showed the specific overexpression profile and the potential application of THBS1 as an ACLF pathogenesis-related biomarker.

### Functional correlations between THBS1, the immune response and cellular apoptosis

Functional analysis of the genes involved in immune- and apoptosis-related biological processes that were significantly differentially expressed in the rat livers of the ACLF group compared with the LC and NC groups showed that *Thbs1* was among the top genes counted for either immune-related (Fig. 3F) or apoptosis-related (Fig. 3G) biological functions. As shown in Fig. 3H, I, serum THBS1 was positively correlated with the immune-mediated cytokines and apoptotic markers detected in liver tissues taken from various ACLF disease stage, highlighting the potential of THBS1 as a disease development-related marker. Full lists of the significant DEGs related to immune mediator and apoptotic marker gene annotations in the enriched biological processes of the ACLF rat liver transcriptome are provided in Additional file 1: Tables S7-S9.

**Fig. 4:**
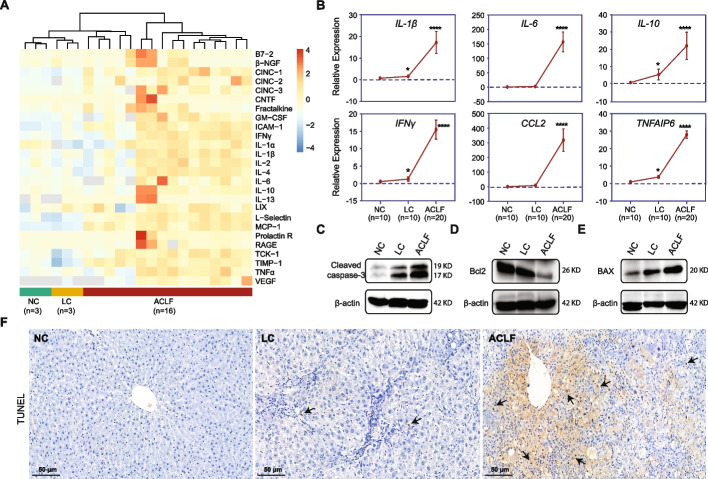
Validation of the inflammatory response and apoptosis as key features in ACLF development. **A** Heatmap of serum ELISA quantification of immunoinflammatory mediators in rats at different disease stages (ACLF = 16; LC = 3; NC = 3). **B** Gene profile validation of certain hepatic inflammatory mediators and immune chemokines in liver tissues collected from rats at different disease stages (ACLF = 20; LC = 10; NC = 10). Data are represented as the mean ± SD. * *P* < 0.05, **** *P* < 0.0001 *vs.* NC, *Gapdh* was used as a reference control gene. **C**-**E** Immunoblotting analysis of hepatocellular apoptosis-related markers; β-actin was used as a loading control. **F** Representative images of the TUNEL assay highlighting the presence of apoptotic hepatocytes (black arrow) in liver tissues collected from rats at different disease stages

Immune target expression levels were elevated in the ACLF stage, as confirmed by Quantibody ® Rat Cytokine Array 3 (RayBiotech, China) using serum from ACLF rats (Fig. 4A). Moreover, the expression profiles of randomly selected hepatic immune-related genes validated the significant upregulation of the inflammatory mediators *Il-6*, *Il-1β*, *Il-10*, *Ifnγ*, *Ccl2* and *Tnfaip6* (*P* < 0.0001) in the ACLF group compared to either the NC or LC group (Fig. 4B). As expected, hepatocyte death parameter analysis indicated the significant overexpression of apoptosis-related proteins, as detected by immunoblotting. These immunoblots displayed a substantial upregulation of cleaved caspase-3 expression (Fig. 4C), especially in the ACLF group. The same expression patterns were observed for other apoptosis-related signaling proteins, among which proapoptotic BAX expression was upregulated (Fig. 4D) while antiapoptotic Bcl-2 expression was downregulated (Fig. 4E). These findings were further fortified by a terminal dUTP nick-end labeling (TUNEL) assay (Abcam, UK), as significant widespread cellular apoptosis and hepatic tissue damage were manifested in the ACLF group compared to the NC group, while LC rats showed a small number of apoptotic cells (Fig. 4F). Collectively, these findings illustrate a mutual relationship between THBS1 expression and the key features of ACLF pathophysiology, inflammation and apoptosis.

### THBS1 is a potential biomarker involved in liver failure pathogenesis

**Fig. 5:**
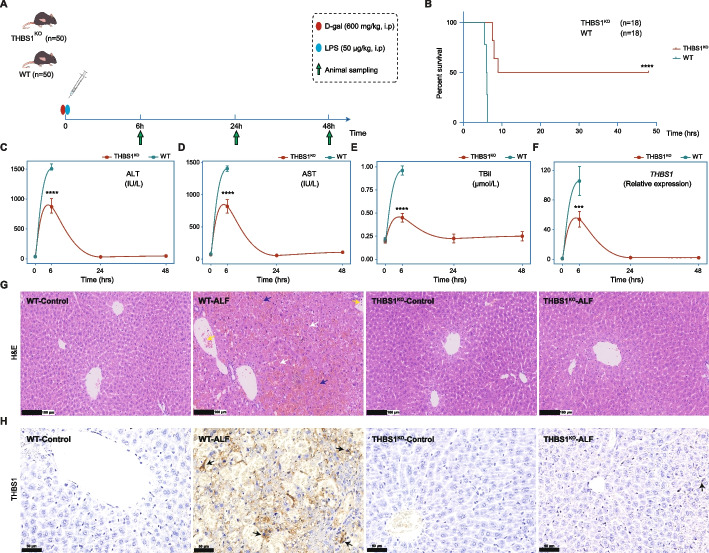
Hepatocyte-specific THBS1 knockout protects against D-gal/LPS-mediated liver failure in mice. **A** Schematic diagram representing ALF model establishment. **B** Kaplan–Meier survival analysis of mice during the ALF period. The survival of WT and hepatocyte-specific THBS1^KO^ mice was observed for 48 h., *n* = 18/group. **C**-**E** Temporal changes in serum ALT, AST and TBil levels. Data are represented as the mean ± SD, *n* = 10 for each time point. **** *P* < 0.0001 *vs.* WT. **F** Expression profile of *Thbs1* in liver tissues. Data are represented as the mean ± SD, *n* = 10 for each time point. *** *P* < 0.001 *vs.* WT; *Gapdh* was used as a reference control gene. **G** Representative histopathological analysis, in which WT mice showed inflammatory cells and inflammatory infiltration (yellow arrow), massive bleeding and injury (purple arrow), hepatocyte death and hepatocellular structural loss (white arrow), and **H** THBS1 immunohistochemistry in liver tissues (black arrow) collected from mice at the normal control and D-gal/LPS-treated WT and hepatocyte-specific THBS1^KO^ mice

To validate our findings regarding the potential role of THBS1 in liver failure and its ability to predict disease severity and outcome, we further assessed the influence of THBS1 profile using an ALF model generated in both hepatocyte-specific THBS1^KO^ and WT mice, as illustrated in Fig. 5A. The D-gal/LPS combination could induce ALF in animals, closely resembling the clinical syndrome, mimicking the systemic inflammatory responses and leading to multiorgan failure [28, 29]. Interestingly, as shown in Fig. 5B, the survival rate of THBS1^KO^ mice following ALF was 55.6% (*n* = 10/18) within 48 h, whereas the WT mortality rate within 7 h was 100% (*n* = 18/18). Levels of serum biochemical indices ALT, AST and TBil, representing liver functions, showed significant increases in the WT group 6 h after liver failure establishment, while the THBS1^KO^ group measurements were significantly lower (*P* < 0.0001) and gradually returned to near-normal levels at 24 h following drugs administration (Fig. 5C-E).

As displayed in Fig. 5F, the hepatic *Thbs1* expression profile revealed significant repression in THBS1^KO^ mice compared to their WT littermates. Histopathological examination of liver sections showed severe and extensive damage in WT-ALF mice, featuring severe hepatocellular necrosis, inflammatory infiltration, bleeding and apoptosis. In contrast, the THBS1^KO^ group revealed almost complete protection against D-gal/LPS-induced liver failure (Fig. 5G). In line with these findings, IHC staining showed upregulated THBS1 protein expression from the control to the ALF state in the WT group, while a nearly complete absence of THBS1 expression was observed in the THBS1^KO^ group following ALF establishment, as illustrated in Fig. 5H.

**Fig. 6:**
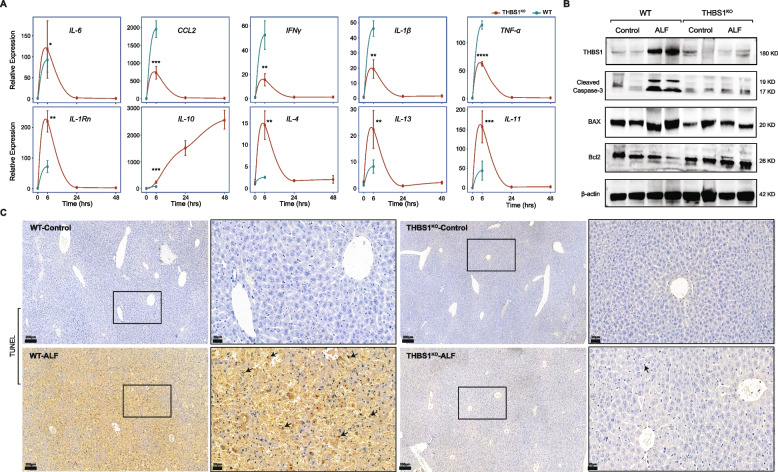
THBS1 deficiency alleviates D-gal/LPS-induced liver failure via anti-inflammatory enhancement and suppression of apoptosis. **A** Changes in hepatic expression of different inflammatory target genes following D-gal/LPS treatment in both WT and THBS1^KO^ mice. Data are represented as the mean ± SD, *n* = 10 for each time point. * *P* < 0.05, ** *P* < 0.01, *** *P* < 0.001, **** *P* < 0.0001 *vs.* WT; *Gapdh* was used as a reference control gene. **B** Representative western immunoblotting of THBS1 and major key targets of the apoptotic cascade in liver tissues collected from WT and THBS1^KO^ mice; β-actin was used as an internal control. **C** D-gal/LPS coadministration caused massive hepatocyte apoptosis (black arrow), characterized by the obvious positive TUNEL staining in the WT mice, whereas THBS1^KO^ prevented the apoptosis-induction ability of the D-gal/LPS

Subsequently, we investigated the changes in immune-inflammatory mediators, as indicated in Fig. 6A, in which THBS1^KO^ mice showed substantial repression of inflammatory cytokines such as *Il-6*, *Ccl2*, *Ifnγ*, *Il-1β*, *Tnfα* and *Il-1rn*. Surprisingly, anti-inflammatory mediators (*Il-10*, *Il-4*, *Il-11* and *Il-13*) were significantly overexpressed (*P* < 0.01) in the THBS1^KO^ liver tissues relative to WT liver tissues. These outcomes strengthened our hypothesis that THBS1 expression is correlated with inflammation, as knocking out THBS1 significantly attenuates the inflammatory responses following liver failure.

To confirm that THBS1 deletion improved mouse survival through inhibition of the hepatocellular apoptotic cascade, we further analyzed THBS1 and apoptotic protein expression using immunoblotting. As a key indicator of cellular apoptosis, the protein levels of THBS1, cleaved caspase-3, and the proapoptotic protein BAX were extensively reduced in THBS1^KO^ mice, highlighting the potential protective capabilities against apoptosis, whereas their corresponding bands were obviously increased in the WT mice following ALF induction (Fig. 6B). Positive TUNEL staining were seen in the WT-ALF liver tissues, while THBS1^KO^-ALF mice showed valuable protection against hepatocellular apoptosis (Fig. 6C). Taken together, diminished THBS1 expression simultaneously enhanced anti-inflammatory properties, promoted hepatic cell protection, and improved liver functions.

## Discussion

ACLF has been a topic of interest for researchers due to its severe incidence rate, rapid disease progression and high mortality [30]. The recognition of ACLF has become more perceptive in recent years and achieved a status of universal acceptance [31–34]. Single molecules and combination of cytokines or chemokines were studied as potential ACLF biomarkers and showed promising results in predicting outcomes [35–37]; however, they were identified based on conventional technologies, which unfortunately could not be sufficiently translated to modern clinical applications. At present, many studies on ACLF are based on alcoholic liver disease and hepatitis C in Western countries, but in Asia-Pacific region, the main etiology is HBV-ACLF [15, 17], in which most of the HBV-ACLF biomarker researches and mechanisms comes from small samples and/or single centers, which limits their future applications. At the same time, there is still a relative lack of research on prognostic molecular targets and their specific pathophysiological mechanisms.

Being the eminent ACLF pathological mechanism, the identification of reliable targets related to immune-metabolism disorder is critical for facilitating timely intensive clinical interventions [38]. Previously, we reported serum levels of certain molecules could predict the onset and severity of HBV-ACLF [39, 40]. Although some of the analyzed biomarkers linked to HBV-ACLF pathogenesis in plasma could have stemmed from other affected organs, the detection of liver-specific biomarkers is somewhat limited by the fact that liver tissues are scarce because of the risk of massive bleeding in HBV-ACLF patients [41]. Previously reported biomarker candidates of HBV-ACLF emerged as a single comparison without optimal prediction of patients’ prognosis [42, 43]. In this study, which was based on the COSSH multicenter cohort, we investigated THBS1 as a potential biomarker and predictor of HBV-ACLF pathogenesis, disease severity and outcome. Through comprehensive analyses with various methods, external cohort validation and development of two liver failure animal models, we finally characterized THBS1 as an important target and potential novel biomarker involved in ACLF pathogenesis. Interestingly, the robust performance of THBS1 was equivalent to the classic scores for predicting ACLF prognosis, whereas the plasma THBS1 cut-off value that stratified patients with low- and high-risk might facilitate the clinical transition of THBS1 as non-invasive tool for ACLF disease progression.

Inflammation and apoptosis are decisive events in the incidence and progression of ACLF [44, 45]. Hepatic inflammasome-induced interleukins (especially IL-1α and IL-1β) are viewed as an important driving force for the development of fatal ACLF [46]. We discovered that THBS1 was significantly correlated with inflammation-related and apoptosis-related processes. THBS1 is recognized as a crucial factor in immune activation and inflammatory response. Overexpression of THBS1 has been documented in conditions associated with tissue damage and inflammation. In the progression of ACLF, elevated THBS1 levels could contribute to liver dysfunction, intensifying hepatic damage and cellular death through the activation of key pathways, such as TGF-β1 and NF-κB, thereby precipitating liver failure. Furthermore, THBS1 can stimulate the up-regulation of IL-1β, IL-6, and TNF-α in immune cells, potentially regulating cell differentiation and apoptosis by activating inflammatory cells [47]. Researchers were able to verify the involvement of THBS1 in various tissue injuries and inflammatory disorders [48–51], mainly through interactions with multiple targets and receptors, such as TGF-β, CD36, integrins and CD47 [11, 52]. In line with this evidence, Starlinger et al. highlighted the effects of THBS1 on liver resection and regeneration abilities, as THBS1 is considered a valuable and clinically relevant predictor of postoperative liver dysfunction and poor clinical outcome after liver resection [53]. Moreover, our results highlighted the significant positive correlations between plasma THBS1 levels and the biochemical indices ALT and γ-GT, the clinical manifestations of liver failure. Both ALT and γ-GT have long been considered as biomarkers reflecting liver disease severity and associated with increased risk of metabolic syndrome and mortality [54]. These findings further support the role of immune-metabolism disorder in the HBV-ACLF pathogenesis previously reported by our group [7, 22]. Overall, THBS1 may further aggravate liver failure by promoting hepatic inflammatory responses and inducing hepatocyte apoptosis. With these multiple lines of evidence, THBS1 has emerged as a new biomarker for predicting ACLF short-term mortality, indicated by a significantly higher AUROC, emphasizing the possible combination of THBS1 with the existing scores for predicting severity could improve the predictive accuracy of ACLF short-term mortality.

In the absence of specific HBV-CLF animal models [55], we applied our previously established ACLF rat model [22], to further assess the potential key role of THBS1 in ACLF pathogenesis. The THBS1 biomarker identified from the human PBMC transcriptome in the COSSH multicenter cohort was externally validated in the serum and liver tissues of ACLF rats, which further confirmed the functional association between THBS1, the immune response and cellular apoptosis. Previously, THBS1 was shown to regulate immune activation, enhance the inflammatory response and accelerate fibrosis [56, 57]. Generally, thrombospondins family are induced in sites of tissue damage or active remodeling, and positively involved in the regulation of cellular responses to injury [58, 59]. THBS1 can modulate inflammation through interaction with the major anti-inflammatory cytokines [60], as blocking THBS1 expression not only prevented inflammatory exacerbation but also was linked to augmentation of anti-inflammatory responses in THBS1^KO^ mice. Similar results were also found in high-fat diet-fed THBS1-deficient mice [61], as well as ureteric obstruction model, which showed blockade of inflammatory lesions and lower renal interstitial inflammation [62]. Interestingly, Min-DeBartolo et al. revealed that THBS1 null mice exhibited a decrease in serum lipid and inflammatory marker levels and hepatic fibrosis in nonalcoholic steatohepatitis [63], but the detailed molecular mechanism of THBS1 regulation of inflammatory response remains to be elucidated.

**Fig. 7:**
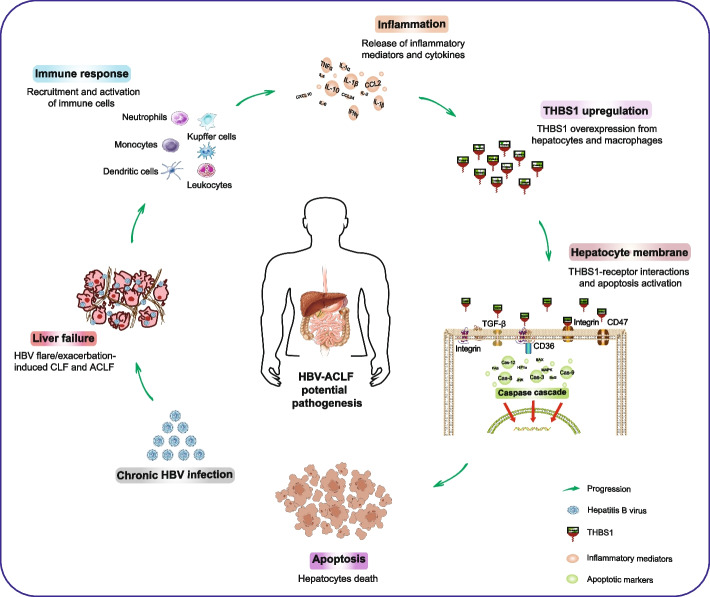
Proposed mechanism of THBS1 in HBV-ACLF pathogenesis. A scheme representing the potential role of THBS1 in HBV-ACLF pathophysiology. HBV exacerbation triggers an immune response, and the subsequent hepatic inflammasome activation promotes THBS1 upregulation from hepatocytes and Kupffer cells. THBS1 activates the caspase cascade, mediated by the interaction between THBS1 and its possible receptors and targets (CD36, TGF-β, CD47, integrins) on the surface of the hepatocyte membrane, which generate hepatocellular apoptosis and promote the liver failure

Many studies have linked THBS1 to the activation of numerous processes that impact cellular apoptosis [64–66]. Our current findings showed upregulation of hepatic THBS1 expression, which was positively associated with hepatocyte apoptosis, denoting that THBS1 promotes cellular death through the induction of apoptotic pathways. In line with this evidence, a significant reduction in hepatic apoptosis, associated with less injury and liver tissue damage, was observed in THBS1^KO^ mice following intoxication. Moreover, in vitro/in vivo experiments revealed that THBS1 selectively triggers cellular apoptosis via sequential provocation of the caspase family [67, 68]. However, in-depth verification is still required to reveal the outcomes of THBS1^KO^ in the dynamic disease course of ACLF. Based on our findings, given the significant correlations of THBS1 with several inflammation- and apoptosis-related genes, we proposed a hypothetical mechanism of THBS1 involvement in ACLF pathogenesis (Fig. 7), which could provide a theoretical foundation and technical support for prospective studies.

Nevertheless, our study is associated with shortcomings, including the study population is based on single precipitant (HBV); however, some prognostic features and disease outcome could vary in ACLF patients with other etiologies as alcoholic cirrhosis, AIH and DILI. Comprehensive investigation of the role of potential confounders that could interfere with THBS1 in predicting the HBV-ACLF outcome also needed to be explored. Second, current lack of universally accepted HBV-based ACLF animal model that represent the disease manifestations, despite the successfulness of our ACLF rat model to resemble the clinical spectrum of the disease. Regardless the shared clinical symptoms between ALF and ACLF [69], which was previously confirmed even at the monocyte transcriptome level [70], we cannot exclude the possibility of distinct differences in the disease entity between ACLF and ALF in our animal models, as mice were insensitive to porcine serum in establishing an immune-based ACLF [71]. If such model is established, the genuine protective capacity of THBS1 knockout could be examined. Third, in light with above findings, a comprehensive exploration of the THBS1 intricate mechanism in the ACLF pathogenesis is still necessary to supplement, and in-depth validations will be conducted in near future with suitable investigations, although it is unlikely to change the overall conclusions of this study.

## Conclusions

In summary, THBS1 could be used as a new biomarker of ACLF pathogenesis, prediction of disease severity and short-term outcome. This study characterized THBS1 as robust ACLF disease-related biomarker, which has great importance in providing clinicians with new targets for improving diagnosis and treatment strategies and reducing the high mortality rate of ACLF. Fully uncovering the comprehensive mechanisms of THBS1 in ACLF pathogenesis is still of utmost importance, particularly in a non-HBV-ACLF population, dynamic longitudinal cohort and in suitable ACLF animal models.

### Supplementary Information

**Additional file 1: Table S1.** Clinical characteristics of enrolled subjects from the COSSH prospective multicenter cohort. **Table S2.** Clinical characteristics of ACLF patients in the ELISA validation group from the COSSH prospective multicenter cohort. **Table S3.** Primers used for real-time PCR. **Table S4.** Antibodies used for immunoblotting and immunohistochemistry. **Table S5.** Clinical characteristics of ACLF patients with different THBS1 relative expression in the validation group. **Table S6.** Clinical characteristics of ACLF patients in the low-risk group and high-risk group of the ELISA validation cohort. **Table S7.** The enriched biological processes for the top 200 genes which were both differentially expressed in the comparisons of ACLF *vs*. LC and ACLF *vs*. NC. **Table S8.** The immune-related biological processes for top 200 DEGs which were both differentially expressed in the comparisons of ACLF *vs*. LC and ACLF *vs*. NC. **Table S9.** The apoptosis-related biological processes for top 200 DEGs which were both differentially expressed in the comparisons of ACLF *vs*. LC and ACLF *vs*. NC.

**Additional file 2: Supplementary Methods.** Supplementary methods.

**Additional file 3: Data File.** Supplementary original western blots.

**Additional file 4: Fig S1.** Correlations between *THBS1* gene with inflammatory and apoptotic markers in the human PBMC transcriptome.

## Data Availability

All data associated with this study are presented within the article or the supplementary materials. Sequencing reads are available in the NCBI Sequence Read Archive database (https://www.ncbi.nlm.nih.gov/sra) with the accession numbers PRJNA713912 and PRJNA548207. Other raw data are available from the corresponding author upon reasonable request.

## Abbreviations

ACLF: Acute-on-chronic liver failure
ALB: Albumin
ALF: Acute liver failure
AUROC: Area under the receiver operating characteristic curve
CANONIC: Chronic Liver Failure (CLIF) Consortium Acute-on-Chronic Liver Failure in Cirrhosis
CHB: Chronic hepatitis B
COSSH: Chinese Group on the Study of Severe Hepatitis B
DEGs: Differentially expressed genes
D-gal: D-galactosamine
GAPDH: Glyceraldehyde-3-phosphate dehydrogenase
HBV: Hepatitis b virus
HBV-ACLF: Hepatitis b virus-related acute-on-chronic liver failure
IHC: Immunohistochemistry
LC: Liver cirrhosis
LPS: Lipopolysaccharide
NC: Normal control
NIH: National Institute of Health
PBMCs: Peripheral blood mononuclear cells
THBS1: Thrombospondin 1
THBS1^KO^: Thrombospondin 1 knockout
TUNEL: Terminal dUTP nick-end labeling
WT: Wild-type
