# Supplementary material for: Thrombospondin 1 enhances systemic inflammation and disease severity in acute-on-chronic liver failure
Source: BMC Med. 2024 Mar 5;22:95. doi: 10.1186/s12916-024-03318-x (PMC10913480; doi:10.1186/s12916-024-03318-x)
Supplement: Supplementary file 1 — Additional file 1: Table S1. Clinical characteristics of enrolled subjects from the COSSH prospective multicenter cohort. Table S2. Clinical characteristics of ACLF patients in the ELISA validation group from the COSSH prospective multicenter cohort. Table S3. Primers used for real-time PCR. Table S4. Antibodies used for immunoblotting and immunohistochemistry. Table S5. Clinical characteristics of ACLF patients with different THBS1 relative expression in the validation group. Table S6. Clinical characteristics of ACLF patients in the low-risk group and high-risk group of the ELISA validation cohort. Table S7. The enriched biological processes for the top 200 genes which were both differentially expressed in the comparisons of ACLF vs. LC and ACLF vs. NC. Table S8. The immune-related biological processes for top 200 DEGs which were both differentially expressed in the comparisons of ACLF vs. LC and ACLF vs. NC. Table S9. The apoptosis-related biological processes for top 200 DEGs which were both differentially expressed in the comparisons of ACLF vs. LC and ACLF vs. NC. [file 12916_2024_3318_MOESM1_ESM.pdf]

ADDITIONAL FILE 1

Thrombospondin 1 enhances systemic inflammation and disease severity in acute-on-chronic liver failure

Hozeifa Mohamed Hassan[†], Xi Liang[†], Jiaojiao Xin[†], Yingyan Lu, Qun Cai, Dongyan Shi, Keke Ren, Jun Li, Qi Chen, Jiang Li, Peng Li, Beibei Guo, Hui Yang, Jinjin Luo, Heng Yao, Xingping Zhou, Wen Hu, Jing Jiang[*], Jun Li[*]

Contents

- **Table S1**
- **Table S2**
- **Table S3**
- **Table S4**
- **Table S5**
- **Table S6**
- **Table S7**
- **Table S8**
- **Table S9**

Table S1. Clinical characteristics of all enrolled subjects

Characteristics	ACLF (n=130)	LC (n=70)	CHB (n=70)	NC (n=60)
Age (yrs.)	44.5 [36.2, 55.0]	46.0 [40.0, 52.5]	43.5 [38.0, 50.0]	46.5 [33.8, 53.0]
Male (%)	86.2% (112)	74.3% (52)	77.1% (54)	56.7% (34) ***
**HBV DNA level (IU/mL)**		Significance†	Significance†	
≤ 2×10^2	5.4% (7)	91.4% (64)	81.4% (57)	NA
$2\times10^2 – 2\times10^6$	66.9% (87)	8.6% (6)	12.9% (9)	NA
> 2×10^6	27.7% (36)	0.0% (0)	5.7% (4)	NA
**Laboratory data**				
Alanine aminotransferase (U/L)	252.0 [93.0, 592.0]	24.5 [18.0, 32.0] ***	23.2 [17.0, 29.8] ***	19.0 [14.0, 25.0] ***
Aspartate aminotransferase (U/L)	147.5 [101.3, 290.8]	23.5 [19.3, 29.8] ***	22.0 [20.0, 26.8] ***	20.0 [18.0, 25.0] ***
Albumin (g/dL)	31.5 [28.5, 33.8]	47.6 [45.6, 48.8] ***	48.7 [46.5, 51.1] ***	47.7 [46.1, 49.1] ***
Total bilirubin (μmol/L)	352.5 [262.9, 437.9]	13.2 [11.0, 17.0] ***	13.0 [9.2, 16.0] ***	11.0 [8.5, 14.0] ***
Alkaline phosphatase (U/L)	137.0 [110.5, 166.0]	70.0 [58.3, 90.0] ***	64.5 [50.5, 79.8] ***	67.00 [55.0, 84.0] ***
γ-Glutamyl transpeptidase (U/L)	77.8 [56.0, 115.4]	22.0 [17.0, 37.8] ***	20.5 [13.0, 32.8] ***	19.0 [12.8, 27.0] ***
Creatinine (μmol/L)	67.0 [58.0, 80.8]	70.5 [62.7, 81.0]	75.0 [65.0, 85.0]	70.5 [57.7, 81.0]
Sodium (mmol/L)	137.0 [135.0, 139.0]	141.0 [140.0, 142.0] ***	141.0 [140.0, 143.0] ***	141.0 [140.0, 143.0] ***
White blood cell count (10^9/L)	7.6 [6.1, 10.3]	5.1 [4.0, 6.3] ***	5.5 [4.7, 6.3] ***	6.1 [5.4, 6.9] ***

Characteristics	ACLF (n=130)	LC (n=70)	CHB (n=70)	NC (n=60)
Hemoglobin (g/L)	125.0 [116.2, 136.0]	153.0 [142.5, 158.8] ***	151.0 [140.0, 162.0] ***	149.0 [136.8, 160.0] ***
Hematocrit (%)	35.6 [32.5, 38.9]	45.5 [42.9, 47.3] ***	44.5 [41.2, 46.9] ***	44.1 [40.7, 47.6] ***
Platelet count (10^9/L)	112.0 [74.0, 145.0]	155.5 [117.0, 207.8] ***	178.9 ± 63.3 ***	218.5 [188.5, 257.2] ***
INR	2.3 [1.8, 3.0]	1.0 [1.0, 1.1] ***	0.97 [0.94, 1.02] ***	NA
Alpha fetoprotein	88.8 [34.8, 235.3]	2.2 [1.8, 2.8] ***	2.5 [1.9, 3.4] ***	2.8 [2.0, 4.0] ***
**Organ failure (%)**				
Liver	94.6% (123)	NA	NA	NA
Coagulation	42.3% (55)	NA	NA	NA
Kidneys	6.2% (8)	NA	NA	NA
Brain	7.7% (10)	NA	NA	NA
Lungs	1.5% (2)	NA	NA	NA
Circulation	0.8% (1)	NA	NA	NA
**Severity score**				
COSSH-ACLF IIs	7.3 [6.7, 7.8]	NA	NA	NA
COSSH-ACLFs	6.2 [5.7, 6.8]	NA	NA	NA

Characteristics	ACLF (n=130)	LC (n=70)	CHB (n=70)	NC (n=60)
CLIF-C ACLFs	42.0 [37.3, 47.1]	NA	NA	NA
MELDs	24.6 [20.5, 28.3]	NA	NA	NA
MELD-Nas	25.7 [22.0, 29.5]	NA	NA	NA
CTP	10.0 [9.0, 11.0]	5.0 [5.0, 5.0]	5.0 [5.0, 5.0]	NA
**Transplant-free mortality rate (number of deceased patients)** ‡				
28-day	33.0% (37)	NA	NA	NA
90-day	46.0% (47)	NA	NA	NA

NOTE. Data are presented as the means ± SDs, medians with [p25, p75], or percentages (numbers of patients). ACLF, acute-on-chronic liver failure; LC, liver cirrhosis; CHB, chronic hepatitis B; NC, normal control; COSSH-ACLFs, COSSH-ACLF score; CLIF-C ACLFs, CLIF Consortium ACLF score; MELDs, Model for End-stage Liver Disease score; MELD-Nas, MELD-sodium score; CTP, Child-Turcotte-Pugh score.

‡Twelve patients with ACLF underwent liver transplantation and were considered lost to follow-up in the mortality rate calculation. Six patients with ACLF were lost to the 28-day follow-up analysis, and 16 patients were lost to the 90-day follow-up analysis.

† *P* value (<0.001) for comparisons of the HBV DNA distribution between the LC/CHB/NC and ACLF groups.

*** *P*<0.001 for comparisons between the LC/CHB/NC and ACLF groups.

Table S2. Clinical characteristics of ACLF patients in the ELISA validation group

Characteristics	All HBV-ACLF (n = 198)	Non-Survivor† (n = 91)	Survivor† (n = 107)
Age (yrs.)	48.3±11.9	52.7±11.3	44.6±11.2
Male (%)	81.3% (161)	80.2% (73)	82.2% (88)
**HBV DNA level (IU/mL)**			
$\leq 2\times10^2$	5.6% (11)	4.4% (4)	8.4% (9)
$2\times10^2 - 2\times10^6$	65.6% (130)	62.6% (57)	66.4% (71)
$> 2\times10^6$	28.8% (57)	33.0% (30)	25.2% (27)
**Laboratory data**			
Alanine aminotransferase (U/L)	224.0 [90.0, 443.0]	218.0 [91.0, 476.0]	244.0 [82.0, 435.0]
Aspartate aminotransferase (U/L)	130.5 [78.8, 277.5]	147.0 [86.0, 323.0]	130.0 [75.0, 216.0]
Albumin (g/dL)	30.7 [28.2, 33.0]	30.0 [27.7, 32.6]	31.1 [28.6, 34.1] *
Total bilirubin (µmol/L)	343.6 [277.7, 427.6]	398.4 [307.5, 452.3]	306.4 [253.5, 378.0] ***
Alkaline phosphatase (U/L)	143.5 [120.0, 176.3]	142.0 [118.0, 179.0]	146.0 [121.0, 176.0]
γ-Glutamyl transpeptidase (U/L)	76.0 [49.0, 120.5]	68.0 [48.0, 114.0]	77.0 [50.0, 123.0]
Creatinine (µmol/L)	61.5 [54.0, 76.3]	63.0 [54.0, 85.0]	60.0 [53.0, 71.0] **

Characteristics	All HBV-ACLF (n = 198)	Non-Survivor† (n = 91)	Survivor† (n = 107)
Serum urea (mmol/L)	4.3 [3.2, 6.2]	5.1 [3.6, 7.6]	3.9 [2.9, 5.2] ***
Triglycerides (mmol/L)	1.2 [0.9, 1.5]	1.0 [0.8, 1.3]	1.3 [1.0, 1.8] ***
Total cholesterol (mmol/L)	2.0 [1.6, 2.6]	1.8 [1.5, 2.3]	2.2 [1.8, 2.7] **
Sodium (mmol/L)	138.0 [136.0, 140.0]	137.0 [135.0, 139.0]	138.0 [136.0, 140.0]
White blood cell count (10^9/L)	7.5 [5.9, 9.6]	7.8 [6.3, 10.3]	6.9 [5.0, 9.4] **
Neutrophils (10^9/L)	5.3 [3.6, 7.1]	5.7 [4.3, 8.2]	4.5 [3.1, 6.8] ***
Hemoglobin (g/L)	122.0 [107.0, 137.0]	121.0 [107.0, 136.0]	123.0 [108, 138.0]
Hematocrit (%)	33.8 [29.9, 39.1]	33.2 [29.7, 38.1]	34.1 [30.5, 39.5]
Platelet count (10^9/L)	100.5 [69.0, 136.3]	91.0 [69.0, 124.0]	110.0 [70.0, 149] *
INR	2.1 [1.8, 2.7]	2.5 [2.0, 3.2]	1.9 [1.6, 2.3] ***
Fibrinogen (g/L)	1.3 [1.0, 1.6]	1.2 [0.9, 1.5]	1.4 [1.1, 1.7] **
Prothrombin time (s)	23.6 [19.9, 29.1]	28.7 [22.7, 34.0]	21.0 [18.6, 25.2] ***
D-dimer (µg/L)	2315.0 [1098.0, 4228.0]	3496.0 [1840.0, 5031.0]	1742.0 [751.0, 3290.0] ***
Alpha fetoprotein	93.5 [30.0, 261.3]	88.6 [23.8, 222.7]	116.7 [30.1, 294.6]
Ferritin (µg/L)	2664.0 [1689.0, 4789.0]	3333.0 [2050.0, 5922.0]	2347.0 [1286.0, 4024.0] **

Characteristics	All HBV-ACLF (n = 198)	Non-Survivor† (n = 91)	Survivor† (n = 107)
**Organ failure (%)**			
Liver	98.5% (195)	97.8% (89)	99.1% (106)
Coagulation	34.9% (69)	55.0% (50)	17.8% (19) ***
Kidneys	4.0% (8)	8.8% (8)	0.0% (0) ***
Brain	9.6% (19)	17.6% (16)	2.8% (3) ***
Lungs	1.5% (3)	3.3% (3)	0.0% (0)
Circulation	3.0% (6)	5.5% (5)	0.9% (1)
**Severity score**			
COSSH-ACLFs	6.2 [5.7, 6.9]	6.8 [6.3, 7.6]	5.8 [5.4, 6.2] ***
COSSH-ACLF IIs	7.2 [6.6, 8.0]	8.0 [7.3, 8.5]	6.7 [6.3, 7.1] ***
CLIF-C ACLFs	42.5 [37.4, 48.2]	47.7 [43.1, 51.3]	38.7 [34.3, 42.4] ***
MELDs	26.6 [24.3, 29.3]	29.0 [26.6, 32.7]	25.0 [23.2, 27.0] ***
MELD-Nas	27.2 [25.2, 30.1]	29.6 [27.1, 32.9]	25.8 [23.9, 27.3] ***
CTP	11.0 [10.0, 12.0]	12.0 [11.0, 12.0]	11.0 [10.0, 11.0] ***

Characteristics	All HBV-ACLF (n = 198)	Non-Survivor† (n = 91)	Survivor† (n = 107)
**ACLF grade**			
ACLF-1	62.6% (124)	40.7% (37)	81.3% (87) **
ACLF-2	28.3% (56)	40.7% (37)	17.8% (19) **
ACLF-3	9.1% (18)	18.6% (17)	0.9% (1) ***
**Transplant-free mortality rate (number of deceased patients)**			
28-day	33.3% (66)	72.5% (66)	0.0% (0) ***
90-day	46.0% (91)	100.0% (91)	0.0% (0) ***

NOTE. Data are presented as the means ± SDs, medians with [p25, p75], or percentages (numbers of patients). ACLF, acute-on-chronic liver failure; COSSH-ACLFs, Chinese Group on the Study of Severe Hepatitis B-ACLF score; COSSH-ACLF IIs, COSSH-ACLF II score; CLIF-C ACLFs, Chronic Liver Failure- Consortium ACLF score; MELDs, model for end-stage liver disease score; MELD-Nas, MELD-sodium score; CTP, child-turcotte-pugh score.

† Non-survivor, HBV-ACLF patients who died within 90 days; Survivor, HBV-ACLF patients who survived 90 days.
**P* value (<0.05), ***P* value (<0.01) and ****P* value (<0.001) for comparisons between non-survivor and survivor group.

Table S3. Primers used for real-time PCR

Gene	Primer orientation	Nucleotide sequence	NCBI Gene ID number
hGAPDH	forward	5’-CTCTCTGCTCCTCCTGTTCG-3’	2597
	reverse	5’-ACGACCAAATCCGTTGACTC-3’	
hTHBS1	forward	5’-AGGCATGTTCCAGTTTCACC-3’	
	reverse	5’-GCTGGCACCACCTTTATTGT-3’	7057
rGAPDH	forward	5’-ATGGAGAAGGCTGGGGCTCACCT-3’	24383
	reverse	5’-AGCCCTTCCACGATGCCAAAGTTGT-3’	
rTHBS1	forward	5’-TAGCTGGAAATGTGGTGCGT-3’	445442
	reverse	5’-TCAGCTTCACATTGCCTGTC-3’	
rCCL2	forward	5’-ATGCAGTTAATGCCCCACTC-3’	24770
	reverse	5’-TTCCTTATTGGGGTCAGCAC-3’	
rIL-10	forward	5’- CCTGCTCTTACTGGCTGGAG -3’	25325
	reverse	5’-TGTCCAGCTGGTCCTTCTTT -3’	
rIL-6	forward	5’-AGAGACTTCCAGCCAGTTGC-3’	24498
	reverse	5’-AGTCTCCTCTCCGGACTTGT-3’	
rIL-1β	forward	5’-CCTGTTCTTTGAGGCTGACA-3’	24494
	reverse	5’-GCTGTGAGATTTGAAGCTGGA-3’	
rINFγ	forward	5’-ACAACCCACAGATCCAGCAC-3’	25712
	reverse	5’-CCAGAATCAGCACCGACTCC-3’	
rTNFAIP6	forward	5’-AAGCAGCCAGAAAGATTGGA -3’	84397
	reverse	5’-TTCGGGTTGTAGCAATAGGC -3’	
mGAPDH	forward	5’-CATCACTGCCACCCAGAAGACTG-3’	21825
	reverse	5’-ATGCCAGTGAGCTTCCCGTTCAG-3’	
mTHBS1	forward	5’-GGTAGCTGGAAATGTGGTGCGT-3’	14433
	reverse	5’-GCACCGATGTTCTCCGTTGTGA-3’	
mCcl2	forward	5’-GCTACAAGAGGATCACCAGCAG-3’	20296
	reverse	5’-GTCTGGACCCATTCCTTCTTGG-3’	

Gene	Primer orientation	Nucleotide sequence	NCBI Gene ID number
mIL-6	forward	5’-CTGCAAGAGACTTCCATCCAG-3’	16193
	reverse	5’-AGTGGTATAGACAGGTCTGTTGG-3’	
mIL-1β	forward	5’-TGGACCTTCCAGGATGAGGACA-3’	16176
	reverse	5’-GTTCATCTCGGAGCCTGTAGTG-3’	
mINFγ	forward	5’-CAGCAACAGCAAGGCGAAAAAGG-3’	15978
	reverse	5’-TTTCCGCTTCCTGAGGCTGGAT-3’	
mTNFAIP6	forward	5’-GGATGCCTATTGCTACAACCCAC-3’	21930
	reverse	5’-CTTGAGCCGAATGTGCCAGTAG-3’	
mTNFα	forward	5’-GGTGCCTATGTCTCAGCCTCTT-3’	21926
	reverse	5’-GCCATAGAACTGATGAGAGGGAG-3’	
mIL-10	forward	5’-CGGGAAGACAATAACTGCACCC-3’	16153
	reverse	5’-CGGTTAGCAGTATGTTGTCCAGC-3’	
mIL-1Rn	forward	5’-TGTGCCTGTCTTGTGCCAAGTC-3’	16181
	reverse	5’-GCCTTTCTCAGAGCGGATGAAG-3’	
mIL-4	forward	5’-ATCATCGGCATTTTGAACGAGGTC-3’	16189
	reverse	5’-ACCTTGGAAGCCCTACAGACGA-3’	
mIL-11	forward	5’-CTGACGGAGATCACAGTCTGGA-3’	16156
	reverse	5’-GGACATCAAGTCTACTCGAAGCC-3’	
mIL-13	forward	5’-AACGGCAGCATGGTATGGAGTG-3’	6163
	reverse	5’-TGGGTCCTGTAGATGGCATTGC-3’	

NOTE. *h*, human; *m*, mouse; *r*, rat.

Table S4. Antibodies for Western immunoblotting and immunohistochemistry

**Primary antibodies**		
Antibody	Catalog number	Company
THBS1	ab1823	Abcam, Cambridge, UK
ALB	HPA031025	Sigma–Aldrich, MO, USA
CD86	ab53004	Abcam, Cambridge, UK
BAX	14796	Cell Signaling Technology (CST), MA, USA
Bcl2	sc-7382	Santa Cruz, CA, USA
Cleaved caspase 3	9661	Cell Signaling Technology (CST) MA, USA
β-actin	Ab8226	Abcam, Cambridge, UK
**Secondary antibodies**		
Antibody	Catalog number	Company
Goat anti-mouse	ab6789	Abcam, Cambridge, UK
Goat anti-rabbit	ab6721	Abcam, Cambridge, UK

Table S5. Clinical characteristics of ACLF patients with different THBS1 expression in the validation group

Characteristics	Group 0<br>THBS1<median (3.5)<br>n = 49	Group 1<br>THBS1>median (3.5)<br>n = 49
**Age (yrs.)**	45.5±10.6	47.7±13.6
**Male (No.)**	85.7% (42)	92.8% (45)
**HBV-DNA level (IU/ml)**		
2×10^2 - 2×10^4	30.6% (15)	22.5% (11)
2×10^4 - 2×10^6	40.8% (20)	36.7% (18)
> 2×10^6	28.6% (14)	40.8% (20)
**Laboratory data**		
Alanine aminotransferase (U/L)	225.0 [87.0, 546.0]	368.0 [178.0, 773.0]
Aspartate aminotransferase (U/L)	148.0 [84.0, 290.0]	187.0 [117.0, 368.0]
Albumin (g/dL)	32.0 [28.0, 35.0]	31.5 [28.5, 33.2]
Total bilirubin (μmol/l)	342.0 [245.0, 432.0]	344.0 [261.0, 405.3]
Alkaline phosphatase (U/L)	133.0 [100.0, 162.0]	137.0 [112.0, 162.0]
γ-Glutamyl transpeptidase (U/L)	67.0 [38.0, 108.0]	76.0 [47.0, 117.0]**
Creatinine (μmol/L)	63.0 [55.0, 72.0]	69.0 [59.0, 91.0]**
Sodium (mmol/L)	137.0 [135.0, 139.0]	137.0 [135.0, 141.0]
White blood cell count (10^9/L)	7.6 [6.1, 9.7]	7.50 [6.2, 10.4]
Hemoglobin (g/L)	126.0 [117.0, 136.0]	125.0 [117.0, 140.0]
Hematocrit (%)	36.6 [33.2, 38.7]	35.6 [33.0, 39.70]
Platelet count (10^9/L)	106.0 [80.0, 172.0]	116.0 [72.0, 133.0]
INR	2.1 [1.7, 2.7]	2.4 [1.8, 2.9]
Alpha fetoprotein	92.4 [33.1, 231.5]	86.4 [30.6 235.3]
**Organ failure (No.)**		
Liver	91.8% (45)	93.9% (46)

Characteristics	Group 0<br>THBS1<median (3.5)<br>n = 49	Group 1<br>THBS1>median (3.5)<br>n = 49
Coagulation	30.6% (15)	49.0% (24)
Kidneys	0.0% (0)	4.1% (2)
Brain	4.1% (2)	8.2% (4)
Lungs	0.0% (0)	2.0% (1)
Circulation	0.0% (0)	2.0% (1)
**ACLF grade**		
ACLF-1	73.5% (36)	51.0% (25)
ACLF-2	22.4% (11)	40.8% (20)
ACLF-3	4.1% (2)	8.2% (4)
**Severity score**		
COSSH-ACLF IIs	7.1 [6.6, 7.5]	7.3 [6.7, 8.1]*
COSSH-ACLFs	6.0 [5.6, 6.7]	6.3 [5.8, 6.9]
CLIF-C ACLFs	40.5 [37.3, 45.1]	41.7 [38.7, 47.2]
MELD-Na	25.4 [20.7, 26.9]	25.9 [22.6, 31.7]
MELD	22.5 [20.2, 26.1]	25.5 [21.6, 30.8]*
CTP	9.0 [8.0, 10.0]	10.0 [9.0, 11.0]*
**Transplant-free mortality (No. deceased patients)** †		
28-day	17.5% (7)	45.0% (18)*
90-day	27.7% (10)	58.3% (21)*

NOTE. Data are expressed as the mean ± standard deviation (SD), median (p25, p75) or percentage (number of patients).
COSSH-ACLF IIs, COSSH-ACLF II score; COSSH-ACLFs, COSSH-ACLF score; CLIF-C ACLFs, CLIF Consortium ACLF score; MELDs, Model for End-stage Liver Disease score; MELD- Nas, MELD-sodium score; CTP, Child-Turcotte-Pugh score; THBS1, thrombospondin 1.
†Twelve patients with ACLF underwent liver transplantation and were considered lost to follow-up in the mortality rate calculation. Six patients with ACLF were lost to the 28-day follow-up analysis, and 14 patients were lost to the 90-day follow-up analysis.
*P <0.05 and **P <0.01 for comparisons between the groups.

Table S6. Clinical characteristics of ACLF patients in the low-risk group and high-risk group of the ELISA validation cohort

Characteristics	Low-risk group n = 75	High-risk group n = 123
**Age (yrs.)**	50.3±12.0	48.2±12.2
**Male (No.)**	78.7% (59)	82.9% (102)
**HBV-DNA level (IU/ml)**		
2×10^2 - 2×10^4	37.3% (28)	36.6% (45)
2×10^4 - 2×10^6	44.0% (33)	34.1% (42)
$> 2\times10^6$	18.7% (14)	29.3% (36)
**Laboratory data**		
Alanine aminotransferase (U/L)	152.0 [55.0, 318.0]	250.0 [100.0, 476.0]*
Aspartate aminotransferase (U/L)	114.0 [67.0, 211.0]	130.0 [80.0, 294.0]
Albumin (g/dL)	30.6 [27.9, 33.8]	30.5 [28.3, 33.2]
Total bilirubin (μmol/l)	310.0 [252.1, 416.5]	366.1 [282.0, 428.9]
Alkaline phosphatase (U/L)	129.0 [109.0, 173.0]	143.0 [122.0, 174.0]
γ-Glutamyl transpeptidase (U/L)	68.0 [39.0, 109.0]	79.0 [54.0, 122.0]*
Creatinine (μmol/L)	65.0 [58.0, 80.0]	60.0 [52.0, 75.0]
Sodium (mmol/L)	138.0 [135.0, 140.0]	138.0 [136.0, 140.0]
White blood cell count (10^9/L)	6.50 [4.5, 9.1]	7.7 [6.1, 9.6]
Hemoglobin (g/L)	112.0 [103.0, 133.0]	125.0 [110.0, 139.0]*
Hematocrit (%)	31.5 [29.6, 38.1]	34.6 [30.8, 39.1]
Platelet count (10^9/L)	76 [55.0, 117.0]	113.0 [78.0, 141.0]***
INR	2.24 [1.79, 2.67]	2.10 [1.80, 2.64]
Alpha fetoprotein	104.1 [29.1, 309.8]	90.5 [33.7, 257.2]
**Organ failure (No.)**		
Liver	96.0% (72)	100.0% (123)

Characteristics	Low-risk group<br>n = 75	High-risk group<br>n = 123
Coagulation	40.0% (30)	32.5% (40)
Kidneys	6.7% (5)	1.6% (2)
Brain	9.3% (7)	8.9% (11)
Lungs	1.3% (1)	0.8% (1)
Circulation	5.3% (4)	1.6% (2)
**ACLF grade**		
ACLF-1	60.0% (45)	61.0% (75)
ACLF-2	32.0% (24)	30.1% (37)
ACLF-3	8.0% (6)	8.9% (11)
**Severity score**		
COSSH-ACLF IIs	7.3 [6.8, 8.1]	7.1 [6.5, 8.0]*
COSSH-ACLFs	6.4 [5.7, 6.9]	6.2 [5.7, 6.8]
CLIF-C ACLFs	43.3 [38.0, 49.1]	42.5 [36.6, 48.2]
MELD-Na	28.5 [25.2, 30.8]	27.0 [25.4, 29.9]
MELD	27.0 [24.1, 30.0]	26.1 [24.3, 28.9]
CTP	11.0 [10.0, 12.0]	11.0 [10.0, 12.0]
**Transplant-free mortality (No. deceased patients)**		
28-day	10.7% (8)	46.3% (57)**
90-day	18.7% (14)	58.5% (72)**

NOTE. Data are expressed as the mean ± standard deviation (SD), median (p25, p75) or percentage (number of patients).

COSSH-ACLF IIs, COSSH-ACLF II score; COSSH-ACLFs, COSSH-ACLF score; CLIF-C ACLFs, CLIF Consortium ACLF score; MELDs, Model for End-stage Liver Disease score; MELD- Nas, MELD-sodium score; CTP, Child-Turcotte-Pugh score; THBS1, thrombospondin 1. $^{*}P$ <0.05, $^{**}P$ <0.01 and $^{***}P$ <0.001 for comparisons between the groups.

Table S7. The enriched biological processes for the top 200 genes which were both differentially expressed in the comparisons of ACLF vs. LC and ACLF vs. NC

GO ID	GO Term	Term PValu	Associated Genes Found
GO:0000186	activation of MAPKK activity	0.03	[CARTPT, GRM1, TNF]
GO:0046847	filopodium assembly	0.03	[CCR7, DNM3, MRAP]
GO:0048747	muscle fiber development	0.03	[KEL, KLHL40, WFIKKN2]
GO:0033555	multicellular organismal response to stress	0.01	[EDNRB, MAPK8IP2, PENK, THBS1]
GO:0050435	amyloid-beta metabolic process	0.02	[ACE, MGAT3, TNF]
GO:0033574	response to testosterone	0.02	[ACOD1, SPP1, THBS1]
GO:0070664	negative regulation of leukocyte proliferation	0.01	[CCL8, FCGR2B, GPNMB, IL10]
GO:0097306	cellular response to alcohol	0.02	[CCL7, PTGER4, RAD51, TNC]
GO:1901889	negative regulation of cell junction assembly	0.00	[ACE, DLEC1, THBS1, TNF]
GO:0034142	toll-like receptor 4 signaling pathway	0.01	[ACOD1, RAB7B, TNIP3]
GO:1904645	response to amyloid-beta	0.00	[FCGR2B, MMP13, MMP3, RAMP3]
GO:2000242	negative regulation of reproductive process	0.00	[LIF, RRAD, SULF1, WT1]
GO:0034637	cellular carbohydrate biosynthetic process	0.02	[B3GNT9, HAS1, LALBA, PHKG1]
GO:0001836	release of cytochrome c from mitochondria	0.03	[IL6, MFF, SFN]
GO:0005179	hormone activity	0.00	[CARTPT, GAST, GPNMB, INHBB, PENK, PNOC, UTS2]
GO:0010469	regulation of signaling receptor activity	0.00	[ADRA2A, CCL2, FCGR2B, IL10, MAPK8IP2, NPTX1, RAMP3, SERPINE1, WFIKKN2]
GO:0035914	skeletal muscle cell differentiation	0.04	[ANKRD2, EGR2, KLHL40]
GO:0015298	solute:cation antiporter activity	0.01	[SLC8A2, SLC9B1, SLC9B2]
GO:0022617	extracellular matrix disassembly	0.01	[ADAMTS4, IL6, MMP13, MMP3]
GO:0030728	ovulation	0.00	[INHBB, PTGS2, TNFAIP6]
GO:0030858	positive regulation of epithelial cell differentiation	0.00	[ETV2, LIF, SERPINE1, SFN]
GO:0031644	regulation of nervous system process	0.00	[CARTPT, EDNRB, EGR2, GRM1, IL10, IL6, NPTX1, SLC8A2]
GO:0032570	response to progesterone	0.00	[ACOD1, FOSB, FOSL1, THBS1]
GO:0042743	hydrogen peroxide metabolic process	0.03	[DUOXA2, HBZ, MMP3]
GO:0002251	organ or tissue specific immune response	0.01	[C17orf99, GCNT3, IL6]
GO:0002381	immunoglobulin production involved in immunoglobulin mediated immune response	0.03	[C17orf99, GCNT3, IL10]
GO:0044321	response to leptin	0.00	[FGF23, INHBB, NR4A3]
GO:0044320	cellular response to leptin stimulus	0.00	[FGF23, INHBB, NR4A3]
GO:0030219	megakaryocyte differentiation	0.02	[H4C1, NR4A3, RAB7B, THBS1]
GO:0045652	regulation of megakaryocyte differentiation	0.01	[H4C1, NR4A3, RAB7B, THBS1]
GO:0046683	response to organophosphorus	0.00	[FOSB, FOSL1, PENK, PTGS2, SELL, WT1]
GO:0014074	response to purine-containing compound	0.00	[ACOD1, FOSB, FOSL1, PENK, PTGS2, SELL, WT1]
GO:0031960	response to corticosteroid	0.00	[ACOD1, FOSB, FOSL1, IL10, IL6, PTGS2, TNF, TPH2, ZFP36L2]
GO:0051384	response to glucocorticoid	0.00	[ACOD1, FOSB, FOSL1, IL10, IL6, PTGS2, TNF, TPH2, ZFP36L2]
GO:0051966	regulation of synaptic transmission, glutamatergic	0.00	[CCL2, GRM1, MAPK8IP2, PTGS2, TNF]
GO:0035249	synaptic transmission, glutamatergic	0.01	[CCL2, GRM1, MAPK8IP2, PTGS2, TNF]
GO:0070542	response to fatty acid	0.02	[CCR7, PTGER4, PTGS2, TNC]
GO:0034694	response to prostaglandin	0.00	[CCR7, PTGER4, TNC]
GO:0070741	response to interleukin-6	0.01	[C1QTNF4, FGF23, IL6]
GO:0071354	cellular response to interleukin-6	0.01	[C1QTNF4, FGF23, IL6]
GO:0071774	response to fibroblast growth factor	0.00	[CCL2, FGF23, FLRT2, SULF1, THBS1, TNC, ZFP36L2]
GO:0044344	cellular response to fibroblast growth factor stimulus	0.00	[CCL2, FGF23, FLRT2, SULF1, THBS1, ZFP36L2]
GO:0008652	cellular amino acid biosynthetic process	0.00	[ACOD1, BCAT1, NAT8L, PLOD2, PYCR1]
GO:1901607	alpha-amino acid biosynthetic process	0.01	[ACOD1, BCAT1, PLOD2, PYCR1]
GO:0043902	positive regulation of multi-organism process	0.01	[EDNRB, INHBB, IQCF1]
GO:2000243	positive regulation of reproductive process	0.01	[EDNRB, INHBB, IQCF1, WT1]
GO:0007218	neuropeptide signaling pathway	0.00	[CARTPT, GPNMB, GPR84, LTB4R, PENK, PNOC]
GO:0005184	neuropeptide hormone activity	0.01	[CARTPT, PENK, PNOC]
GO:0008237	metallopeptidase activity	0.00	[ACE, ADAM12, ADAMTS4, KEL, MEP1B, MMP13, MMP3, NAALADL1, WFIKKN2]
GO:0004222	metalloendopeptidase activity	0.00	[ADAM12, ADAMTS4, KEL, MEP1B, MMP13, MMP3, WFIKKN2]
GO:0014909	smooth muscle cell migration	0.04	[ACE, NR4A3, SERPINE1]
GO:0014910	regulation of smooth muscle cell migration	0.03	[ACE, NR4A3, SERPINE1]
GO:0002286	T cell activation involved in immune response	0.00	[CLEC4D, FCGR2B, IL6, PTGER4, TNFSF18]
GO:0002292	T cell differentiation involved in immune response	0.03	[CLEC4D, IL6, PTGER4]
GO:0043062	extracellular structure organization	0.00	[ADAM12, ADAMTS4, FLRT2, HAS1, IL6, MMP13, MMP3, P3H4, SERPINE1, SPP1, SULF1, THBS1, TNC, TNF, VCAN, WT1]
GO:0030198	extracellular matrix organization	0.00	[ADAM12, ADAMTS4, FLRT2, HAS1, IL6, MMP13, MMP3, P3H4, SERPINE1, SPP1, SULF1, THBS1, TNC, TNF, VCAN, WT1]
GO:0007566	embryo implantation	0.00	[ACOD1, LIF, PTGS2, SPP1]
GO:0001893	maternal placenta development	0.01	[LIF, PTGS2, SPP1]
GO:0046697	decidualization	0.00	[LIF, PTGS2, SPP1]
GO:0002701	negative regulation of production of molecular mediator of immune response	0.01	[FCGR2B, IL10, TNF]
GO:0050766	positive regulation of phagocytosis	0.04	[CCL2, FCGR2B, TNF]
GO:0031663	lipopolysaccharide-mediated signaling pathway	0.03	[ACOD1, CCL2, TNF]
GO:0019233	sensory perception of pain	0.00	[CCL2, EDNRB, GRM1, IL10, PENK, PTGS2]
GO:0051931	regulation of sensory perception	0.01	[EDNRB, GRM1, IL10]
GO:0051930	regulation of sensory perception of pain	0.01	[EDNRB, GRM1, IL10]
GO:1902895	positive regulation of pri-miRNA transcription by RNA polymerase II	0.00	[FOSL1, IL10, TNF, WT1]
GO:0061614	pri-miRNA transcription by RNA polymerase II	0.00	[FOSL1, IL10, TNF, WT1]
GO:1902893	regulation of pri-miRNA transcription by RNA polymerase II	0.00	[FOSL1, IL10, TNF, WT1]
GO:0045428	regulation of nitric oxide biosynthetic process	0.03	[IL10, PTGS2, TNF]
GO:1903670	regulation of sprouting angiogenesis	0.01	[BMPER, IL10, PTGS2, THBS1]
GO:1903672	positive regulation of sprouting angiogenesis	0.01	[BMPER, IL10, PTGS2]
GO:0030545	receptor regulator activity	0.00	[C10orf99, C17orf99, C1QTNF4, CARTPT, CCL2, CCL22, CCL7, CCL8, EBI3, FGF23, FLRT2, GAST, GPNMB, IL10, IL6, INHBB, LIF, MRAP, PENK, PNOC, SPP1, TNF, TNFSF18, UTS2, WFIKKN2]
GO:0048018	receptor ligand activity	0.00	[C10orf99, C17orf99, C1QTNF4, CARTPT, CCL2, CCL22, CCL7, CCL8, EBI3, FGF23, FLRT2, GAST, GPNMB, IL10, IL6, INHBB, LIF, PENK, PNOC, SPP1, TNF, TNFSF18, UTS2]
GO:0030546	signaling receptor activator activity	0.00	[C10orf99, C17orf99, C1QTNF4, CARTPT, CCL2, CCL22, CCL7, CCL8, EBI3, FGF23, FLRT2, GAST, GPNMB, IL10, IL6, INHBB, LIF, PENK, PNOC, SPP1, TNF, TNFSF18, UTS2]
GO:0005125	cytokine activity	0.00	[C10orf99, C17orf99, C1QTNF4, CCL2, CCL22, CCL7, CCL8, EBI3, IL10, IL6, INHBB, LIF, SPP1, TNF, TNFSF18]
GO:0061005	cell differentiation involved in kidney development	0.03	[ACTA2, LIF, WT1]
GO:0032835	glomerulus development	0.03	[ACTA2, SULF1, WT1]
GO:0035850	epithelial cell differentiation involved in kidney development	0.02	[ACTA2, LIF, WT1]
GO:0072207	metanephric epithelium development	0.00	[LIF, SOAT1, WT1]
GO:0097722	sperm motility	0.00	[CFAP65, IQCF1, SEPTIN12, SLC9B1, SLC9B2, TCTE1, TEKT3]
GO:0001539	cilium or flagellum-dependent cell motility	0.00	[CFAP65, IQCF1, SEPTIN12, SLC9B1, SLC9B2, TCTE1, TEKT3]
GO:0030317	flagellated sperm motility	0.00	[CFAP65, IQCF1, SEPTIN12, SLC9B1, SLC9B2, TCTE1, TEKT3]
GO:0060285	cilium-dependent cell motility	0.00	[CFAP65, IQCF1, SEPTIN12, SLC9B1, SLC9B2, TCTE1, TEKT3]
GO:1904377	positive regulation of protein localization to cell periphery	0.03	[RAMP3, STAC2, TNF]
GO:1905477	positive regulation of protein localization to membrane	0.00	[CDK5R1, MFF, RAMP3, SFN, STAC2, TNF]
GO:1903078	positive regulation of protein localization to plasma membrane	0.02	[RAMP3, STAC2, TNF]
GO:1904427	positive regulation of calcium ion transmembrane transport	0.04	[JPH2, RAMP3, STAC2]
GO:0060425	lung morphogenesis	0.02	[LIF, TNC, TNF]
GO:0002761	regulation of myeloid leukocyte differentiation	0.01	[CARTPT, FSTL3, LIF, SLC9B2, TNF]
GO:0002763	positive regulation of myeloid leukocyte differentiation	0.02	[LIF, SLC9B2, TNF]
GO:0045670	regulation of osteoclast differentiation	0.01	[CARTPT, FSTL3, SLC9B2, TNF]
GO:0051148	negative regulation of muscle cell differentiation	0.02	[ANKRD2, BHLHE41, TRIM72]
GO:0014902	myotube differentiation	0.00	[ADAM12, ANKRD2, BHLHE41, KEL, KLHL40, TRIM72]
GO:0051154	negative regulation of striated muscle cell differentiation	0.01	[ANKRD2, BHLHE41, TRIM72]
GO:0010830	regulation of myotube differentiation	0.02	[ANKRD2, BHLHE41, TRIM72]
GO:0010832	negative regulation of myotube differentiation	0.00	[ANKRD2, BHLHE41, TRIM72]
GO:0045123	cellular extravasation	0.00	[CCL2, PTGER4, SELE, SELL, TNF]
GO:0061756	leukocyte adhesion to vascular endothelial cell	0.00	[IL6, SELE, SELL, TNF]
GO:0050901	leukocyte tethering or rolling	0.00	[SELE, SELL, TNF]
GO:1904994	regulation of leukocyte adhesion to vascular endothelial cell	0.00	[IL6, SELE, TNF]
GO:1904996	positive regulation of leukocyte adhesion to vascular endothelial cell	0.00	[IL6, SELE, TNF]
GO:0031670	cellular response to nutrient	0.01	[FGF23, PENK, PTGS2, TNC]
GO:0033273	response to vitamin	0.00	[FGF23, PENK, PTGS2, SPP1, TNC]
GO:0033280	response to vitamin D	0.00	[FGF23, PENK, PTGS2, SPP1, TNC]
GO:0071295	cellular response to vitamin	0.00	[FGF23, PENK, TNC]
GO:0071305	cellular response to vitamin D	0.00	[FGF23, PENK, TNC]
GO:0043277	apoptotic cell clearance	0.01	[CCL2, FCN1, THBS1]
GO:1905517	macrophage migration	0.03	[CCL2, EDNRB, THBS1]
GO:0048246	macrophage chemotaxis	0.01	[CCL2, EDNRB, THBS1]
GO:0019934	cGMP-mediated signaling	0.00	[CGAS, EDNRB, THBS1]
GO:1904037	positive regulation of epithelial cell apoptotic process	0.00	[CCL2, IL6, THBS1]
GO:0050680	negative regulation of epithelial cell proliferation	0.00	[CCL2, RUNX3, SFN, SULF1, THBS1, TNF]
GO:0001937	negative regulation of endothelial cell proliferation	0.00	[CCL2, SULF1, THBS1, TNF]
GO:0072577	endothelial cell apoptotic process	0.00	[CCL2, IL10, SERPINE1, THBS1, TNF]
GO:1904035	regulation of epithelial cell apoptotic process	0.00	[CCL2, IL6, SERPINE1, THBS1, TNF]
GO:2000351	regulation of endothelial cell apoptotic process	0.00	[CCL2, SERPINE1, THBS1, TNF]
GO:0034103	regulation of tissue remodeling	0.01	[CARTPT, GPNMB, IL6, SPP1]
GO:0031646	positive regulation of nervous system process	0.04	[CARTPT, EGR2, IL6]
GO:0046850	regulation of bone remodeling	0.02	[CARTPT, IL6, SPP1]
GO:0045453	bone resorption	0.04	[CARTPT, IL6, SPP1]
GO:0045124	regulation of bone resorption	0.01	[CARTPT, IL6, SPP1]
GO:0031102	neuron projection regeneration	0.03	[IL6, SPP1, TNC]
GO:1990868	response to chemokine	0.00	[CCL2, CCL22, CCL7, CCL8, CCR7]
GO:0070098	chemokine-mediated signaling pathway	0.00	[CCL2, CCL22, CCL7, CCL8, CCR7]
GO:1990869	cellular response to chemokine	0.00	[CCL2, CCL22, CCL7, CCL8, CCR7]
GO:0030593	neutrophil chemotaxis	0.01	[CCL2, CCL22, CCL7, CCL8, CCR7]
GO:0070371	ERK1 and ERK2 cascade	0.00	[BMPER, CCL2, CCL22, CCL7, CCL8, CCR7, FGF23, GLIPR2, GPNMB, LIF, PTGER4, RAMP3, TNF, ZFP36L2]
GO:0070374	positive regulation of ERK1 and ERK2 cascade	0.00	[BMPER, CCL2, CCL22, CCL7, CCL8, CCR7, FGF23, GLIPR2, GPNMB, RAMP3, TNF]
GO:0051048	negative regulation of secretion	0.00	[ADRA2A, CARTPT, CCR7, FCGR2B, FGF23, GPNMB, IL10, INHBB, LIF, PTGER4, TNF]
GO:1903531	negative regulation of secretion by cell	0.00	[ADRA2A, CARTPT, CCR7, FCGR2B, FGF23, GPNMB, IL10, INHBB, LIF, PTGER4, TNF]
GO:0046888	negative regulation of hormone secretion	0.00	[ADRA2A, CARTPT, FGF23, GPNMB, INHBB, LIF]
GO:0002792	negative regulation of peptide secretion	0.00	[ADRA2A, CARTPT, CCR7, FCGR2B, IL10, INHBB, PTGER4, TNF]
GO:0050709	negative regulation of protein secretion	0.00	[ADRA2A, CCR7, FCGR2B, IL10, INHBB, PTGER4, TNF]
GO:0050710	negative regulation of cytokine secretion	0.00	[CCR7, FCGR2B, IL10, PTGER4, TNF]
GO:0090278	negative regulation of peptide hormone secretion	0.01	[ADRA2A, CARTPT, INHBB]
GO:0002468	dendritic cell antigen processing and presentation	0.00	[CCR7, FCGR2B, THBS1]
GO:0002577	regulation of antigen processing and presentation	0.00	[CCR7, FCGR2B, THBS1]
GO:0002604	regulation of dendritic cell antigen processing and presentation	0.00	[CCR7, FCGR2B, THBS1]
GO:0032615	interleukin-12 production	0.03	[CCR7, IL10, THBS1]
GO:0002544	chronic inflammatory response	0.00	[IL10, THBS1, TNF]
GO:0032655	regulation of interleukin-12 production	0.02	[CCR7, IL10, THBS1]
GO:0032695	negative regulation of interleukin-12 production	0.00	[CCR7, IL10, THBS1]
GO:1905953	negative regulation of lipid localization	0.01	[IL6, THBS1, TNF]
GO:0043277	apoptotic cell clearance	0.01	[CCL2, FCN1, THBS1]
GO:1904019	epithelial cell apoptotic process	0.00	[CCL2, IL10, IL6, SERPINE1, THBS1, TNF]
GO:1905517	macrophage migration	0.03	[CCL2, EDNRB, THBS1]
GO:0002688	regulation of leukocyte chemotaxis	0.00	[CCL2, CCL7, CCR7, IL6, SERPINE1, THBS1]
GO:0048246	macrophage chemotaxis	0.01	[CCL2, EDNRB, THBS1]
GO:1904035	regulation of epithelial cell apoptotic process	0.00	[CCL2, IL6, SERPINE1, THBS1, TNF]
GO:1904037	positive regulation of epithelial cell apoptotic process	0.00	[CCL2, IL6, THBS1]
GO:1902041	regulation of extrinsic apoptotic signaling pathway via death domain receptors	0.03	[IL6, SERPINE1, THBS1]
GO:0035150	regulation of tube size	0.00	[ACE, ACTA2, ADRA2A, EDNRB, KEL, PTGS2, UTS2]
GO:0006939	smooth muscle contraction	0.00	[ACTA2, ADRA2A, CNN1, EDNRB, PTGS2, SULF1]
GO:0035296	regulation of tube diameter	0.00	[ACE, ACTA2, ADRA2A, EDNRB, KEL, PTGS2, UTS2]
GO:0097746	regulation of blood vessel diameter	0.00	[ACE, ACTA2, ADRA2A, EDNRB, KEL, PTGS2, UTS2]
GO:0006940	regulation of smooth muscle contraction	0.03	[ADRA2A, CNN1, PTGS2]
GO:0097756	negative regulation of blood vessel diameter	0.00	[ACE, ACTA2, ADRA2A, EDNRB, KEL, PTGS2]
GO:0019229	regulation of vasoconstriction	0.03	[ACE, ADRA2A, PTGS2]
GO:0051926	negative regulation of calcium ion transport	0.04	[ADRA2A, PTGS2, RRAD]
GO:0042310	vasoconstriction	0.00	[ACE, ACTA2, ADRA2A, EDNRB, KEL, PTGS2]
GO:0150076	neuroinflammatory response	0.01	[IL6, MMP3, PTGS2, TNF]
GO:0006953	acute-phase response	0.00	[EDNRB, IL6, PTGS2, TNF]
GO:0150077	regulation of neuroinflammatory response	0.00	[IL6, MMP3, PTGS2, TNF]
GO:0002675	positive regulation of acute inflammatory response	0.00	[CCR7, IL6, PTGS2, TNF]
GO:0014015	positive regulation of gliogenesis	0.01	[EGR2, IL6, LIF, TNF]
GO:0007260	tyrosine phosphorylation of STAT protein	0.01	[IL6, LIF, TNF, TNFSF18]
GO:0042509	regulation of tyrosine phosphorylation of STAT protein	0.01	[IL6, LIF, TNF, TNFSF18]
GO:0033138	positive regulation of peptidyl-serine phosphorylation	0.01	[IL6, LIF, PTGS2, RAMP3, TNF]
GO:0042531	positive regulation of tyrosine phosphorylation of STAT protein	0.01	[IL6, LIF, TNF, TNFSF18]

GO:0032350	regulation of hormone metabolic process	0.01	[DUOXA2, GNB3, TNF]
GO:0031650	regulation of heat generation	0.00	[EDNRB, PTGS2, TNF]
GO:0031649	heat generation	0.00	[EDNRB, PTGS2, TNF]
GO:1903426	regulation of reactive oxygen species biosynthetic process	0.02	[DUOXA2, IL10, PTGS2, TNF]
GO:2000379	positive regulation of reactive oxygen species metabolic process	0.00	[ACOD1, DUOXA2, PTGS2, THBS1, TNF]
GO:0045428	regulation of nitric oxide biosynthetic process	0.03	[IL10, PTGS2, TNF]
GO:1903428	positive regulation of reactive oxygen species biosynthetic process	0.02	[DUOXA2, PTGS2, TNF]
GO:0001660	fever generation	0.00	[EDNRB, PTGS2, TNF]
GO:0002673	regulation of acute inflammatory response	0.00	[CCR7, EDNRB, FCGR2B, IL6, PTGS2, TNF]
GO:0006953	acute-phase response	0.00	[EDNRB, IL6, PTGS2, TNF]
GO:0031620	regulation of fever generation	0.00	[EDNRB, PTGS2, TNF]
GO:1905953	negative regulation of lipid localization	0.01	[IL6, THBS1, TNF]
GO:0048659	smooth muscle cell proliferation	0.00	[CNN1, IL10, IL6, NR4A3, PTGS2, THBS1, TNF]
GO:0048660	regulation of smooth muscle cell proliferation	0.00	[CNN1, IL10, IL6, NR4A3, PTGS2, THBS1, TNF]
GO:0048661	positive regulation of smooth muscle cell proliferation	0.00	[IL10, IL6, NR4A3, PTGS2, THBS1, TNF]
GO:0150076	neuroinflammatory response	0.01	[IL6, MMP3, PTGS2, TNF]
GO:0006953	acute-phase response	0.00	[EDNRB, IL6, PTGS2, TNF]
GO:0008625	extrinsic apoptotic signaling pathway via death domain receptors	0.02	[IL6, SERPINE1, THBS1, TNF]
GO:0042108	positive regulation of cytokine biosynthetic process	0.01	[EBI3, IL6, THBS1, TNF]
GO:0150077	regulation of neuroinflammatory response	0.00	[IL6, MMP3, PTGS2, TNF]
GO:0002675	positive regulation of acute inflammatory response	0.00	[CCR7, IL6, PTGS2, TNF]
GO:0043154	negative regulation of cysteine-type endopeptidase activity involved in apoptotic process	0.00	[IL6, PTGS2, SFN, THBS1, TNF]
GO:2000117	negative regulation of cysteine-type endopeptidase activity	0.00	[IL6, PTGS2, SFN, THBS1, TNF]
GO:1905953	negative regulation of lipid localization	0.01	[IL6, THBS1, TNF]
GO:0060688	regulation of morphogenesis of a branching structure	0.03	[IL6, SULF1, TNF]
GO:0010573	vascular endothelial growth factor production	0.01	[IL6, PTGS2, SULF1]
GO:0045598	regulation of fat cell differentiation	0.00	[GNB3, IL6, MEDAG, PTGS2, TNF, ZFP36L2]
GO:0045599	negative regulation of fat cell differentiation	0.01	[IL6, TNF, ZFP36L2]
GO:0061756	leukocyte adhesion to vascular endothelial cell	0.00	[IL6, SELE, SELL, TNF]
GO:0010574	regulation of vascular endothelial growth factor production	0.00	[IL6, PTGS2, SULF1]
GO:0050901	leukocyte tethering or rolling	0.00	[SELE, SELL, TNF]
GO:0010575	positive regulation of vascular endothelial growth factor production	0.00	[IL6, PTGS2, SULF1]
GO:0014009	glial cell proliferation	0.02	[IL6, PENK, TNF]
GO:0032715	negative regulation of interleukin-6 production	0.01	[IL10, IL6, TNF]
GO:1904994	regulation of leukocyte adhesion to vascular endothelial cell	0.00	[IL6, SELE, TNF]
GO:1904996	positive regulation of leukocyte adhesion to vascular endothelial cell	0.00	[IL6, SELE, TNF]
GO:0031650	regulation of heat generation	0.00	[EDNRB, PTGS2, TNF]
GO:0002526	acute inflammatory response	0.00	[CCR7, EDNRB, FCGR2B, IL6, PTGS2, TNF, TREM1]
GO:0031649	heat generation	0.00	[EDNRB, PTGS2, TNF]
GO:0150076	neuroinflammatory response	0.01	[IL6, MMP3, PTGS2, TNF]
GO:0001660	fever generation	0.00	[EDNRB, PTGS2, TNF]
GO:0002673	regulation of acute inflammatory response	0.00	[CCR7, EDNRB, FCGR2B, IL6, PTGS2, TNF]
GO:0006953	acute-phase response	0.00	[EDNRB, IL6, PTGS2, TNF]
GO:0031620	regulation of fever generation	0.00	[EDNRB, PTGS2, TNF]
GO:0150077	regulation of neuroinflammatory response	0.00	[IL6, MMP3, PTGS2, TNF]
GO:0002675	positive regulation of acute inflammatory response	0.00	[CCR7, IL6, PTGS2, TNF]
GO:0033138	positive regulation of peptidyl-serine phosphorylation	0.01	[IL6, LIF, PTGS2, RAMP3, TNF]
GO:0043154	negative regulation of cysteine-type endopeptidase activity involved in apoptotic process	0.00	[IL6, PTGS2, SFN, THBS1, TNF]
GO:2000117	negative regulation of cysteine-type endopeptidase activity	0.00	[IL6, PTGS2, SFN, THBS1, TNF]
GO:0097696	receptor signaling pathway via STAT	0.00	[CCL2, CDK5R1, IL10, IL6, LIF, TNF, TNFSF18]
GO:0002718	regulation of cytokine production involved in immune response	0.02	[IL10, IL6, NR4A3, TNF]
GO:1904892	regulation of receptor signaling pathway via STAT	0.00	[CDK5R1, IL10, IL6, LIF, TNF, TNFSF18]
GO:0007259	receptor signaling pathway via JAK-STAT	0.00	[CCL2, CDK5R1, IL10, IL6, LIF, TNF, TNFSF18]
GO:0032715	negative regulation of interleukin-6 production	0.01	[IL10, IL6, TNF]
GO:1904894	positive regulation of receptor signaling pathway via STAT	0.00	[IL10, IL6, LIF, TNF, TNFSF18]
GO:0046425	regulation of receptor signaling pathway via JAK-STAT	0.00	[CDK5R1, IL10, IL6, LIF, TNF, TNFSF18]
GO:0046427	positive regulation of receptor signaling pathway via JAK-STAT	0.00	[IL10, IL6, LIF, TNF, TNFSF18]
GO:0014015	positive regulation of gliogenesis	0.01	[EGR2, IL6, LIF, TNF]
GO:0007260	tyrosine phosphorylation of STAT protein	0.01	[IL6, LIF, TNF, TNFSF18]
GO:0042509	regulation of tyrosine phosphorylation of STAT protein	0.01	[IL6, LIF, TNF, TNFSF18]
GO:0033138	positive regulation of peptidyl-serine phosphorylation	0.01	[IL6, LIF, PTGS2, RAMP3, TNF]
GO:0042531	positive regulation of tyrosine phosphorylation of STAT protein	0.01	[IL6, LIF, TNF, TNFSF18]
GO:2001024	negative regulation of response to drug	0.00	[IL10, IL6, NR4A3, PYCR1]
GO:2001038	regulation of cellular response to drug	0.00	[IL10, IL6, NR4A3, PYCR1]
GO:1902883	negative regulation of response to oxidative stress	0.00	[IL10, IL6, NR4A3, PYCR1]
GO:2001039	negative regulation of cellular response to drug	0.00	[IL10, IL6, NR4A3, PYCR1]
GO:0031100	animal organ regeneration	0.01	[ACOD1, IL10, IL6, NR4A3]
GO:0071887	leukocyte apoptotic process	0.01	[CCR7, IL10, IL6, NR4A3, PTCRA]
GO:1900408	negative regulation of cellular response to oxidative stress	0.00	[IL10, IL6, NR4A3, PYCR1]
GO:0002718	regulation of cytokine production involved in immune response	0.02	[IL10, IL6, NR4A3, TNF]
GO:1901032	negative regulation of response to reactive oxygen species	0.00	[IL10, IL6, NR4A3, PYCR1]
GO:1903202	negative regulation of oxidative stress-induced cell death	0.00	[IL10, IL6, NR4A3, PYCR1]
GO:0036474	cell death in response to hydrogen peroxide	0.00	[IL10, IL6, NR4A3, PYCR1]
GO:1903205	regulation of hydrogen peroxide-induced cell death	0.00	[IL10, IL6, NR4A3, PYCR1]
GO:2000106	regulation of leukocyte apoptotic process	0.01	[CCR7, IL10, NR4A3, PTCRA]
GO:1903206	negative regulation of hydrogen peroxide-induced cell death	0.00	[IL10, IL6, NR4A3, PYCR1]
GO:0002700	regulation of production of molecular mediator of immune response	0.00	[C17orf99, CGAS, FCGR2B, IL10, IL6, NR4A3, TNF]
GO:0048659	smooth muscle cell proliferation	0.00	[CNN1, IL10, IL6, NR4A3, PTGS2, THBS1, TNF]
GO:2001024	negative regulation of response to drug	0.00	[IL10, IL6, NR4A3, PYCR1]
GO:2001038	regulation of cellular response to drug	0.00	[IL10, IL6, NR4A3, PYCR1]
GO:0042116	macrophage activation	0.00	[FCGR2B, IL10, IL6, THBS1, TNF]
GO:0048660	regulation of smooth muscle cell proliferation	0.00	[CNN1, IL10, IL6, NR4A3, PTGS2, THBS1, TNF]
GO:1902882	regulation of response to oxidative stress	0.00	[IL10, IL6, MMP3, NR4A3, PYCR1, TNF]
GO:1902883	negative regulation of response to oxidative stress	0.00	[IL10, IL6, NR4A3, PYCR1]
GO:2001039	negative regulation of cellular response to drug	0.00	[IL10, IL6, NR4A3, PYCR1]
GO:0036473	cell death in response to oxidative stress	0.00	[IL10, IL6, MMP3, NR4A3, PYCR1]
GO:0031100	animal organ regeneration	0.01	[ACOD1, IL10, IL6, NR4A3]
GO:0048661	positive regulation of smooth muscle cell proliferation	0.00	[IL10, IL6, NR4A3, PTGS2, THBS1, TNF]
GO:0071887	leukocyte apoptotic process	0.01	[CCR7, IL10, IL6, NR4A3, PTCRA]
GO:1900407	regulation of cellular response to oxidative stress	0.00	[IL10, IL6, MMP3, NR4A3, PYCR1, TNF]
GO:1900408	negative regulation of cellular response to oxidative stress	0.00	[IL10, IL6, NR4A3, PYCR1]
GO:1990874	vascular smooth muscle cell proliferation	0.00	[CNN1, IL10, NR4A3, TNF]
GO:0002718	regulation of cytokine production involved in immune response	0.02	[IL10, IL6, NR4A3, TNF]
GO:1901031	regulation of response to reactive oxygen species	0.00	[IL10, IL6, NR4A3, PYCR1, TNF]
GO:1901032	negative regulation of response to reactive oxygen species	0.00	[IL10, IL6, NR4A3, PYCR1]
GO:1903201	regulation of oxidative stress-induced cell death	0.00	[IL10, IL6, MMP3, NR4A3, PYCR1]
GO:1904705	regulation of vascular smooth muscle cell proliferation	0.00	[CNN1, IL10, NR4A3, TNF]
GO:0032715	negative regulation of interleukin-6 production	0.01	[IL10, IL6, TNF]
GO:1903202	negative regulation of oxidative stress-induced cell death	0.00	[IL10, IL6, NR4A3, PYCR1]
GO:1904707	positive regulation of vascular smooth muscle cell proliferation	0.01	[IL10, NR4A3, TNF]
GO:1904894	positive regulation of receptor signaling pathway via STAT	0.00	[IL10, IL6, LIF, TNF, TNFSF18]
GO:0036474	cell death in response to hydrogen peroxide	0.00	[IL10, IL6, NR4A3, PYCR1]
GO:1903205	regulation of hydrogen peroxide-induced cell death	0.00	[IL10, IL6, NR4A3, PYCR1]
GO:0046427	positive regulation of receptor signaling pathway via JAK-STAT	0.00	[IL10, IL6, LIF, TNF, TNFSF18]
GO:1903206	negative regulation of hydrogen peroxide-induced cell death	0.00	[IL10, IL6, NR4A3, PYCR1]
GO:0050818	regulation of coagulation	0.02	[NR4A3, PROCR, SERPINE1, THBS1]
GO:1905953	negative regulation of lipid localization	0.01	[IL6, THBS1, TNF]
GO:0002437	inflammatory response to antigenic stimulus	0.00	[CCR7, FCGR2B, IL10, TNF]
GO:0002700	regulation of production of molecular mediator of immune response	0.00	[C17orf99, CGAS, FCGR2B, IL10, IL6, NR4A3, TNF]
GO:0048659	smooth muscle cell proliferation	0.00	[CNN1, IL10, IL6, NR4A3, PTGS2, THBS1, TNF]
GO:0050819	negative regulation of coagulation	0.02	[PROCR, SERPINE1, THBS1]
GO:0060688	regulation of morphogenesis of a branching structure	0.03	[IL6, SULF1, TNF]
GO:0002701	negative regulation of production of molecular mediator of immune response	0.01	[FCGR2B, IL10, TNF]
GO:0032615	interleukin-12 production	0.03	[CCR7, IL10, THBS1]
GO:0032637	interleukin-8 production	0.01	[FCN1, IL10, SERPINE1, TNF]
GO:0042107	cytokine metabolic process	0.00	[EBI3, IL10, IL6, INHBB, THBS1, TNF]
GO:0042116	macrophage activation	0.00	[FCGR2B, IL10, IL6, THBS1, TNF]
GO:0048660	regulation of smooth muscle cell proliferation	0.00	[CNN1, IL10, IL6, NR4A3, PTGS2, THBS1, TNF]
GO:0051048	negative regulation of secretion	0.00	[ADRA2A, CARTPT, CCR7, FCGR2B, FGF23, GPNMB, IL10, INHBB, LIF, PTGER4, TNF]
GO:1902882	regulation of response to oxidative stress	0.00	[IL10, IL6, MMP3, NR4A3, PYCR1, TNF]
GO:0030595	leukocyte chemotaxis	0.00	[C10orf99, CCL2, CCL22, CCL7, CCL8, CCR7, CH25H, EDNRB, IL10, IL6, SERPINE1, THBS1]
GO:0042089	cytokine biosynthetic process	0.00	[EBI3, IL10, IL6, INHBB, THBS1, TNF]
GO:0071674	mononuclear cell migration	0.00	[CCL2, CCL22, CCL7, CCL8, IL6, SERPINE1, THBS1, TNF]
GO:0072676	lymphocyte migration	0.00	[C10orf99, CCL2, CCL22, CCL7, CCL8, CCR7, CH25H]
GO:0097529	myeloid leukocyte migration	0.00	[CCL2, CCL22, CCL7, CCL8, CCR7, EDNRB, IL6, PTGER4, SERPINE1, THBS1]
GO:0031650	regulation of heat generation	0.00	[EDNRB, PTGS2, TNF]
GO:0002544	chronic inflammatory response	0.00	[IL10, THBS1, TNF]
GO:0002637	regulation of immunoglobulin production	0.00	[C17orf99, CGAS, FCGR2B, IL10, IL6, TNF]
GO:0002685	regulation of leukocyte migration	0.00	[C10orf99, CCL2, CCL7, CCL8, CCR7, IL6, PTGER4, SELE, SERPINE1, THBS1, TNF, TNFSF18]
GO:0002922	positive regulation of humoral immune response	0.00	[ACOD1, CCR7, FCGR2B, TNF]
GO:0010812	negative regulation of cell-substrate adhesion	0.03	[DLEC1, SERPINE1, THBS1]
GO:0031649	heat generation	0.00	[EDNRB, PTGS2, TNF]
GO:0043030	regulation of macrophage activation	0.00	[FCGR2B, IL10, IL6, THBS1]
GO:0045599	negative regulation of fat cell differentiation	0.01	[IL6, TNF, ZFP36L2]
GO:0048661	positive regulation of smooth muscle cell proliferation	0.00	[IL10, IL6, NR4A3, PTGS2, THBS1, TNF]
GO:0050766	positive regulation of phagocytosis	0.04	[CCL2, FCGR2B, TNF]
GO:1900407	regulation of cellular response to oxidative stress	0.00	[IL10, IL6, MMP3, NR4A3, PYCR1, TNF]
GO:1903426	regulation of reactive oxygen species biosynthetic process	0.02	[DUOXA2, IL10, PTGS2, TNF]
GO:1903531	negative regulation of secretion by cell	0.00	[ADRA2A, CARTPT, CCR7, FCGR2B, FGF23, GPNMB, IL10, INHBB, LIF, PTGER4, TNF]
GO:1904019	epithelial cell apoptotic process	0.00	[CCL2, IL10, IL6, SERPINE1, THBS1, TNF]
GO:1990868	response to chemokine	0.00	[CCL2, CCL22, CCL7, CCL8, CCR7]
GO:1990874	vascular smooth muscle cell proliferation	0.00	[CNN1, IL10, NR4A3, TNF]
GO:0002687	positive regulation of leukocyte migration	0.00	[CCL7, CCL8, CCR7, IL6, SELE, SERPINE1, THBS1, TNF, TNFSF18]
GO:0002718	regulation of cytokine production involved in immune response	0.02	[IL10, IL6, NR4A3, TNF]
GO:0002861	regulation of inflammatory response to antigenic stimulus	0.00	[CCR7, FCGR2B, IL10, TNF]
GO:0032655	regulation of interleukin-12 production	0.02	[CCR7, IL10, THBS1]
GO:0032677	regulation of interleukin-8 production	0.01	[FCN1, IL10, SERPINE1, TNF]
GO:0033619	membrane protein proteolysis	0.02	[ADRA2A, IL10, TNF]
GO:0042035	regulation of cytokine biosynthetic process	0.00	[EBI3, IL10, IL6, INHBB, THBS1, TNF]
GO:0045428	regulation of nitric oxide biosynthetic process	0.03	[IL10, PTGS2, TNF]
GO:0090303	positive regulation of wound healing	0.03	[ADRA2A, SERPINE1, THBS1]
GO:1901031	regulation of response to reactive oxygen species	0.00	[IL10, IL6, NR4A3, PYCR1, TNF]
GO:1903428	positive regulation of reactive oxygen species biosynthetic process	0.02	[DUOXA2, PTGS2, TNF]
GO:1904705	regulation of vascular smooth muscle cell proliferation	0.00	[CNN1, IL10, NR4A3, TNF]
GO:1904892	regulation of receptor signaling pathway via STAT	0.00	[CDK5R1, IL10, IL6, LIF, TNF, TNFSF18]
GO:0002548	monocyte chemotaxis	0.00	[CCL2, CCL22, CCL7, CCL8, IL6, SERPINE1]
GO:0048247	lymphocyte chemotaxis	0.00	[C10orf99, CCL2, CCL22, CCL7, CCL8, CH25H]
GO:0097530	granulocyte migration	0.00	[CCL2, CCL22, CCL7, CCL8, CCR7, PTGER4, THBS1]
GO:0001660	fever generation	0.00	[EDNRB, PTGS2, TNF]
GO:0001937	negative regulation of endothelial cell proliferation	0.00	[CCL2, SULF1, THBS1, TNF]
GO:0001953	negative regulation of cell-matrix adhesion	0.01	[DLEC1, SERPINE1, THBS1]
GO:0002688	regulation of leukocyte chemotaxis	0.00	[CCL2, CCL7, CCR7, IL6, SERPINE1, THBS1]
GO:0008625	extrinsic apoptotic signaling pathway via death domain receptors	0.02	[IL6, SERPINE1, THBS1, TNF]
GO:0014009	glial cell proliferation	0.02	[IL6, PENK, TNF]
GO:0031620	regulation of fever generation	0.00	[EDNRB, PTGS2, TNF]
GO:0032695	negative regulation of interleukin-12 production	0.00	[CCR7, IL10, THBS1]
GO:0032715	negative regulation of interleukin-6 production	0.01	[IL10, IL6, TNF]
GO:0032757	positive regulation of interleukin-8 production	0.02	[FCN1, SERPINE1, TNF]
GO:0042036	negative regulation of cytokine biosynthetic process	0.00	[IL10, IL6, INHBB]
GO:0042108	positive regulation of cytokine biosynthetic process	0.01	[EBI3, IL6, THBS1, TNF]
GO:0070098	chemokine-mediated signaling pathway	0.00	[CCL2, CCL22, CCL7, CCL8, CCR7]
GO:0071621	granulocyte chemotaxis	0.00	[CCL2, CCL22, CCL7, CCL8, CCR7, THBS1]

GO:0071675	regulation of mononuclear cell migration	0.02	[SERPINE1, THBS1, TNF]
GO:0072577	endothelial cell apoptotic process	0.00	[CCL2, IL10, SERPINE1, THBS1, TNF]
GO:1904707	positive regulation of vascular smooth muscle cell proliferation	0.01	[IL10, NR4A3, TNF]
GO:1904894	positive regulation of receptor signaling pathway via STAT	0.00	[IL10, IL6, LIF, TNF, TNFSF18]
GO:1990869	cellular response to chemokine	0.00	[CCL2, CCL22, CCL7, CCL8, CCR7]
GO:2000401	regulation of lymphocyte migration	0.03	[C10orf99, CCL2, CCL7]
GO:0002690	positive regulation of leukocyte chemotaxis	0.00	[CCL7, CCR7, IL6, SERPINE1, THBS1]
GO:0002712	regulation of B cell mediated immunity	0.00	[C17orf99, FCGR2B, IL10, TNF]
GO:0002792	negative regulation of peptide secretion	0.00	[ADRA2A, CARTPT, CCR7, FCGR2B, IL10, INHBB, PTGER4, TNF]
GO:0006509	membrane protein ectodomain proteolysis	0.01	[ADRA2A, IL10, TNF]
GO:0008009	chemokine activity	0.00	[C10orf99, CCL2, CCL22, CCL7, CCL8]
GO:0031638	zymogen activation	0.02	[CUZD1, SERPINE1, THBS1]
GO:0046425	regulation of receptor signaling pathway via JAK-STAT	0.00	[CDK5R1, IL10, IL6, LIF, TNF, TNFSF18]
GO:0051043	regulation of membrane protein ectodomain proteolysis	0.00	[ADRA2A, IL10, TNF]
GO:1904035	regulation of epithelial cell apoptotic process	0.00	[CCL2, IL6, SERPINE1, THBS1, TNF]
GO:1904994	regulation of leukocyte adhesion to vascular endothelial cell	0.00	[IL6, SELE, TNF]
GO:0072677	eosinophil migration	0.00	[CCL2, CCL7, CCL8, PTGER4]
GO:0030593	neutrophil chemotaxis	0.01	[CCL2, CCL22, CCL7, CCL8, CCR7]
GO:0046427	positive regulation of receptor signaling pathway via JAK-STAT	0.00	[IL10, IL6, LIF, TNF, TNFSF18]
GO:0048245	eosinophil chemotaxis	0.00	[CCL2, CCL7, CCL8]
GO:0050709	negative regulation of protein secretion	0.00	[ADRA2A, CCR7, FCGR2B, IL10, INHBB, PTGER4, TNF]
GO:0050710	negative regulation of cytokine secretion	0.00	[CCR7, FCGR2B, IL10, PTGER4, TNF]
GO:1904037	positive regulation of epithelial cell apoptotic process	0.00	[CCL2, IL6, THBS1]
GO:1904996	positive regulation of leukocyte adhesion to vascular endothelial cell	0.00	[IL6, SELE, TNF]
GO:0002889	regulation of immunoglobulin mediated immune response	0.00	[C17orf99, FCGR2B, IL10, TNF]
GO:1902041	regulation of extrinsic apoptotic signaling pathway via death domain receptors	0.03	[IL6, SERPINE1, THBS1]
GO:2000351	regulation of endothelial cell apoptotic process	0.00	[CCL2, SERPINE1, THBS1, TNF]
GO:0043154	negative regulation of cysteine-type endopeptidase activity involved in apoptotic process	0.00	[IL6, PTGS2, SFN, THBS1, TNF]
GO:0070374	positive regulation of ERK1 and ERK2 cascade	0.00	[BMPER, CCL2, CCL22, CCL7, CCL8, CCR7, FGF23, GLIPR2, GPNMB, RAMP3, TNF]
GO:1903672	positive regulation of sprouting angiogenesis	0.01	[BMPER, IL10, PTGS2]
GO:2000117	negative regulation of cysteine-type endopeptidase activity	0.00	[IL6, PTGS2, SFN, THBS1, TNF]

Table S8. The immune-related biological processes for top 200 DEGs which were both differentially expressed in the comparisons of ACLF *vs.* LC and ACLF *vs.* NC

GO ID	GO Term	Term PValue	Associated Genes Found
GO:0070664	negative regulation of leukocyte proliferation	0.0142	[CCL8, FCGR2B, GPNMB, IL10]
GO:0002251	organ or tissue specific immune response	0.0091	[C17orf99, GCNT3, IL6]
GO:0002381	immunoglobulin production involved in immunoglobulin mediated immune response	0.0308	[C17orf99, GCNT3, IL10]
GO:0070741	response to interleukin-6	0.0097	[C1QTNF4, FGF23, IL6]
GO:0071354	cellular response to interleukin-6	0.0074	[C1QTNF4, FGF23, IL6]
GO:0002286	T cell activation involved in immune response	0.0048	[CLEC4D, FCGR2B, IL6, PTGER4, TNFSF18]
GO:0002292	T cell differentiation involved in immune response	0.0332	[CLEC4D, IL6, PTGER4]
GO:0002701	negative regulation of production of molecular mediator of immune response	0.0069	[FCGR2B, IL10, TNF]
GO:0002761	regulation of myeloid leukocyte differentiation	0.0085	[CARTPT, FSTL3, LIF, SLC9B2, TNF]
GO:0002763	positive regulation of myeloid leukocyte differentiation	0.0213	[LIF, SLC9B2, TNF]
GO:0061756	leukocyte adhesion to vascular endothelial cell	0.0011	[IL6, SELE, SELL, TNF]
GO:0050901	leukocyte tethering or rolling	0.004	[SELE, SELL, TNF]
GO:1904994	regulation of leukocyte adhesion to vascular endothelial cell	0.003	[IL6, SELE, TNF]
GO:1904996	positive regulation of leukocyte adhesion to vascular endothelial cell	0.0013	[IL6, SELE, TNF]
GO:1905517	macrophage migration	0.0274	[CCL2, EDNRB, THBS1]
GO:0048246	macrophage chemotaxis	0.0145	[CCL2, EDNRB, THBS1]
GO:0002468	dendritic cell antigen processing and presentation	0.0002	[CCR7, FCGR2B, THBS1]
GO:0002577	regulation of antigen processing and presentation	0.0015	[CCR7, FCGR2B, THBS1]
GO:0002604	regulation of dendritic cell antigen processing and presentation	0.0002	[CCR7, FCGR2B, THBS1]
GO:0032615	interleukin-12 production	0.0264	[CCR7, IL10, THBS1]
GO:0002544	chronic inflammatory response	0.0009	[IL10, THBS1, TNF]
GO:0032655	regulation of interleukin-12 production	0.0243	[CCR7, IL10, THBS1]
GO:0032695	negative regulation of interleukin-12 production	0.0009	[CCR7, IL10, THBS1]
GO:0002688	regulation of leukocyte chemotaxis	0.0025	[CCL2, CCL7, CCR7, IL6, SERPINE1, THBS1]
GO:0150076	neuroinflammatory response	0.0062	[IL6, MMP3, PTGS2, TNF]
GO:0150077	regulation of neuroinflammatory response	0.0004	[IL6, MMP3, PTGS2, TNF]
GO:0002675	positive regulation of acute inflammatory response	0.0003	[CCR7, IL6, PTGS2, TNF]
GO:0031649	heat generation	0.0009	[EDNRB, PTGS2, TNF]
GO:0001660	fever generation	0.0002	[EDNRB, PTGS2, TNF]
GO:0002673	regulation of acute inflammatory response	1E-05	[CCR7, EDNRB, FCGR2B, IL6, PTGS2, TNF]
GO:0006953	acute-phase response	0.0035	[EDNRB, IL6, PTGS2, TNF]
GO:0031620	regulation of fever generation	0.0001	[EDNRB, PTGS2, TNF]
GO:0032715	negative regulation of interleukin-6 production	0.0116	[IL10, IL6, TNF]
GO:0002526	acute inflammatory response	0.0003	[CCR7, EDNRB, FCGR2B, IL6, PTGS2, TNF, TREM1]
GO:0002718	regulation of cytokine production involved in immune response	0.0181	[IL10, IL6, NR4A3, TNF]
GO:0002700	regulation of production of molecular mediator of immune response	0.0015	[C17orf99, CGAS, FCGR2B, IL10, IL6, NR4A3, TNF]
GO:0042116	macrophage activation	0.0038	[FCGR2B, IL10, IL6, THBS1, TNF]
GO:0002437	inflammatory response to antigenic stimulus	0.0039	[CCR7, FCGR2B, IL10, TNF]
GO:0032637	interleukin-8 production	0.0123	[FCN1, IL10, SERPINE1, TNF]
GO:0030595	leukocyte chemotaxis	9E-06	[C10orf99, CCL2, CCL22, CCL7, CCL8, CCR7, CH25H, EDNRB, IL10, IL6, SERPINE1, THBS1]
GO:0071674	mononuclear cell migration	9E-06	[CCL2, CCL22, CCL7, CCL8, IL6, SERPINE1, THBS1, TNF]
GO:0072676	lymphocyte migration	0.0002	[C10orf99, CCL2, CCL22, CCL7, CCL8, CCR7, CH25H]
GO:0097529	myeloid leukocyte migration	0.0002	[CCL2, CCL22, CCL7, CCL8, CCR7, EDNRB, IL6, PTGER4, SERPINE1, THBS1]
GO:0002637	regulation of immunoglobulin production	0.0001	[C17orf99, CGAS, FCGR2B, IL10, IL6, TNF]
GO:0002685	regulation of leukocyte migration	3E-06	[C10orf99, CCL2, CCL7, CCL8, CCR7, IL6, PTGER4, SELE, SERPINE1, THBS1, TNF, TNFSF18]
GO:0002922	positive regulation of humoral immune response	3E-05	[ACOD1, CCR7, FCGR2B, TNF]
GO:0043030	regulation of macrophage activation	0.0035	[FCGR2B, IL10, IL6, THBS1]
GO:0002687	positive regulation of leukocyte migration	2E-05	[CCL7, CCL8, CCR7, IL6, SELE, SERPINE1, THBS1, TNF, TNFSF18]
GO:0002861	regulation of inflammatory response to antigenic stimulus	0.0002	[CCR7, FCGR2B, IL10, TNF]
GO:0032677	regulation of interleukin-8 production	0.0085	[FCN1, IL10, SERPINE1, TNF]
GO:0002548	monocyte chemotaxis	8E-05	[CCL2, CCL22, CCL7, CCL8, IL6, SERPINE1]
GO:0048247	lymphocyte chemotaxis	6E-05	[C10orf99, CCL2, CCL22, CCL7, CCL8, CH25H]
GO:0097530	granulocyte migration	0.0012	[CCL2, CCL22, CCL7, CCL8, CCR7, PTGER4, THBS1]
GO:0032757	positive regulation of interleukin-8 production	0.0204	[FCN1, SERPINE1, TNF]
GO:0071621	granulocyte chemotaxis	0.0028	[CCL2, CCL22, CCL7, CCL8, CCR7, THBS1]
GO:0071675	regulation of mononuclear cell migration	0.0195	[SERPINE1, THBS1, TNF]
GO:2000401	regulation of lymphocyte migration	0.0264	[C10orf99, CCL2, CCL7]
GO:0002690	positive regulation of leukocyte chemotaxis	0.0035	[CCL7, CCR7, IL6, SERPINE1, THBS1]
GO:0002712	regulation of B cell mediated immunity	0.0031	[C17orf99, FCGR2B, IL10, TNF]
GO:0072677	eosinophil migration	0.0002	[CCL2, CCL7, CCL8, PTGER4]
GO:0030593	neutrophil chemotaxis	0.0052	[CCL2, CCL22, CCL7, CCL8, CCR7]
GO:0048245	eosinophil chemotaxis	0.0019	[CCL2, CCL7, CCL8]
GO:0002889	regulation of immunoglobulin mediated immune response	0.0031	[C17orf99, FCGR2B, IL10, TNF]

Table S9. The apoptosis-related biological processes for top 200 DEGs which were both differentially expressed in the comparisons of ACLF *vs.* LC and ACLF *vs.* NC

GO ID	GO Term	Term PValue	Associated Genes Found
GO:0043277	apoptotic cell clearance	0.0137	[CCL2, FCN1, THBS1]
GO:1904037	positive regulation of epithelial cell apoptotic process	0.0047	[CCL2, IL6, THBS1]
GO:0072577	endothelial cell apoptotic process	0.0003	[CCL2, IL10, SERPINE1, THBS1, TNF]
GO:1904035	regulation of epithelial cell apoptotic process	0.0019	[CCL2, IL6, SERPINE1, THBS1, TNF]
GO:2000351	regulation of endothelial cell apoptotic process	0.0017	[CCL2, SERPINE1, THBS1, TNF]
GO:0043277	apoptotic cell clearance	0.0137	[CCL2, FCN1, THBS1]
GO:1904019	epithelial cell apoptotic process	0.0009	[CCL2, IL10, IL6, SERPINE1, THBS1, TNF]
GO:1904035	regulation of epithelial cell apoptotic process	0.0019	[CCL2, IL6, SERPINE1, THBS1, TNF]
GO:1904037	positive regulation of epithelial cell apoptotic process	0.0047	[CCL2, IL6, THBS1]
GO:1902041	regulation of extrinsic apoptotic signaling pathway via death domain receptors	0.0332	[IL6, SERPINE1, THBS1]
GO:0008625	extrinsic apoptotic signaling pathway via death domain receptors	0.017	[IL6, SERPINE1, THBS1, TNF]
GO:0043154	negative regulation of cysteine-type endopeptidase activity involved in apoptotic process	0.0029	[IL6, PTGS2, SFN, THBS1, TNF]
GO:0043154	negative regulation of cysteine-type endopeptidase activity involved in apoptotic process	0.0029	[IL6, PTGS2, SFN, THBS1, TNF]
GO:0071887	leukocyte apoptotic process	0.0065	[CCR7, IL10, IL6, NR4A3, PTCRA]
GO:1903202	negative regulation of oxidative stress-induced cell death	0.0027	[IL10, IL6, NR4A3, PYCR1]
GO:0036474	cell death in response to hydrogen peroxide	0.0004	[IL10, IL6, NR4A3, PYCR1]
GO:1903205	regulation of hydrogen peroxide-induced cell death	0.0002	[IL10, IL6, NR4A3, PYCR1]
GO:2000106	regulation of leukocyte apoptotic process	0.0137	[CCR7, IL10, NR4A3, PTCRA]
GO:1903206	negative regulation of hydrogen peroxide-induced cell death	0.0001	[IL10, IL6, NR4A3, PYCR1]
GO:0036473	cell death in response to oxidative stress	0.0033	[IL10, IL6, MMP3, NR4A3, PYCR1]
GO:0071887	leukocyte apoptotic process	0.0065	[CCR7, IL10, IL6, NR4A3, PTCRA]
GO:1903201	regulation of oxidative stress-induced cell death	0.0012	[IL10, IL6, MMP3, NR4A3, PYCR1]
GO:1903202	negative regulation of oxidative stress-induced cell death	0.0027	[IL10, IL6, NR4A3, PYCR1]
GO:0036474	cell death in response to hydrogen peroxide	0.0004	[IL10, IL6, NR4A3, PYCR1]
GO:1903205	regulation of hydrogen peroxide-induced cell death	0.0002	[IL10, IL6, NR4A3, PYCR1]
GO:1903206	negative regulation of hydrogen peroxide-induced cell death	0.0001	[IL10, IL6, NR4A3, PYCR1]
GO:1904019	epithelial cell apoptotic process	0.0009	[CCL2, IL10, IL6, SERPINE1, THBS1, TNF]
GO:0008625	extrinsic apoptotic signaling pathway via death domain receptors	0.017	[IL6, SERPINE1, THBS1, TNF]
GO:0072577	endothelial cell apoptotic process	0.0003	[CCL2, IL10, SERPINE1, THBS1, TNF]
GO:1904035	regulation of epithelial cell apoptotic process	0.0019	[CCL2, IL6, SERPINE1, THBS1, TNF]
GO:1904037	positive regulation of epithelial cell apoptotic process	0.0047	[CCL2, IL6, THBS1]
GO:1902041	regulation of extrinsic apoptotic signaling pathway via death domain receptors	0.0332	[IL6, SERPINE1, THBS1]
GO:2000351	regulation of endothelial cell apoptotic process	0.0017	[CCL2, SERPINE1, THBS1, TNF]
GO:0043154	negative regulation of cysteine-type endopeptidase activity involved in apoptotic process	0.0029	[IL6, PTGS2, SFN, THBS1, TNF]
