# Supplementary material for: Thrombospondin 1 enhances systemic inflammation and disease severity in acute-on-chronic liver failure
Source: BMC Med. 2024 Mar 5;22:95. doi: 10.1186/s12916-024-03318-x (PMC10913480; doi:10.1186/s12916-024-03318-x)
Supplement: Supplementary file 2 — Additional file 2: Supplementary Methods. Supplementary methods. [file 12916_2024_3318_MOESM2_ESM.pdf]

ADDITIONAL FILE 2

Thrombospondin 1 enhances systemic inflammation and disease severity in acute-on-chronic liver failure

Hozeifa Mohamed Hassan[†], Xi Liang[†], Jiaojiao Xin[†], Yingyan Lu, Qun Cai, Dongyan Shi, Keke Ren, Jun Li, Qi Chen, Jiang Li, Peng Li, Beibei Guo, Hui Yang, Jinjin Luo, Heng Yao, Xingping Zhou, Wen Hu, Jing Jiang[*], Jun Li[*]

Contents

- **Supplementary Methods**

Supplementary Methods

Patient criteria

In this study, the participants are from COSSH multicenter cohort, which were recruited consecutively based on the following criteria:

HBV-ACLF: Subjects with HBV-ACLF were identified according to the COSSH-ACLF criteria, as previously reported. The definition implies HBV-ACLF as a complicated syndrome with a high short-term mortality rate that develops in patients with HBV-related chronic liver disease, regardless of the presence of cirrhosis, and it is characterized by acute deterioration of liver function and hepatic and/or extrahepatic organ failure. HBV-ACLF involves three grades: ACLF-1, ACLF-2 and ACLF-3. The ACLF-1 comprises four types of patients: (1) patients presenting with liver failure alone with an international normalized ratio (INR) $\geq$ 1.5 and/or kidney dysfunction and/or hepatic encephalopathy (HE) grade I or II; (2) patients with kidney failure alone; (3) patients with failure of a single organ failure in the coagulation, circulatory or respiratory system and/or kidney dysfunction and/or HE grade I or II and (4) patients with cerebral failure plus kidney dysfunction. ACLF-2 comprises patients with failures in two organ systems, while ACLF-3 comprises patients with failures in three or more organ systems.

LC and CHB: LC was defined as patients with stable compensated cirrhosis, which was diagnosed on the basis of previous liver biopsy results, clinical evidence, laboratory tests, and endoscopic (esophageal and gastric varices) and radiological imaging of portal hypertension and/or liver nodularity, as previously described. The etiology of all patients with cirrhosis was HBV-related. Patients with a history of decompensation (ascites/HE/upper gastrointestinal hemorrhage/bacterial

infection) were excluded. The enrolment criteria for the patients with CHB corresponded to the 2016 American Association for the Study of Liver Diseases (AASLD) guidelines.

Patients in the CHB and LC groups (n = 70, each) were receiving antiviral therapy, and their viral load (HBV-DNA) was controlled. In the ACLF group, all the patients received the necessary measures to control their viral loads. All patients received necessary measures for integrative treatment, including treatment administration for ascites, HE, and bacterial infections, and renal replacement for hepatorenal syndrome.

NC: Normal healthy volunteers presented with normal physical examination (subjects with underlying common diseases, such as diabetes mellitus and hypertension, were excluded).

Blood samples were all collected at the time of admission (within the first 24 h). Relevant clinical data, including demographic data, cirrhosis complications, history of episodes and precipitating events associated with acute deterioration of HBV-related chronic liver disease and ACLF, demographic, laboratory indicators were collected at admission and follow-up data were collected from the electronic data capture system and case report forms. Information regarding liver transplantation and survival time was also collected.

Selection of PBMCs

Liver tissue scarcity has limited the accurate investigation of the pathogenesis of HBV- ACLF, as patients experiencing severe hepatic failure may not be able to undergo a biopsy. Therefore, the use of blood as a surrogate tissue, which can be obtained with a minimally invasive procedure, is an attractive alternative to hepatic biopsies. The PBMC transcriptome can serve as a signature of many diseases, including HBV-ACLF.

Drugs and reagents

Porcine serum (catalog No 26250084) was obtained from Gibco, Grand Island, NY, USA. D-Galactosamine (D-gal, Lot No SLBM3382V, analytical standard ≥99%) and lipopolysaccharide (LPS, Lot No BCBR3574V, derived from Escherichia coli 0128:B12 serotype, source strain CDC 2440-69) were purchased from Sigma–Aldrich (St. Louis, MO, USA). Serum biomarker detection reagents were equipment-based. Pathology-related reagents were supplied by the Department of Pathology, The First Affiliated Hospital, Zhejiang University School of Medicine (Hangzhou, China). Serum levels of THBS1 were measured by ELISA (Lot No ab193716, Abcam, UK for human samples; Lot No abx256081, Abbexa, USA for rat samples).

HBV DNA measurement

HBV DNA levels were quantified by real-time PCR using a COBAS® instrument (Roche, Switzerland) and HBV Test v2.0.

Evaluation of biochemical function

Biochemical tests of liver function were evaluated by an automatic AU5800 clinical chemistry analyzer (Beckman, USA).

Identification of DEGs

Adaptors and low-quality reads were removed using Trimmomatic v0.36 with the default parameters. Paired-end transcriptome sequencing reads from each sample were aligned with a human reference genome (GRCh38.87, for human PBMCs) or aligned with a rat reference genome (Rnor 6.0, for rat liver tissues) using HISAT v2.0.5 with the default parameters. For the PBMC transcriptome, StringTie v1.3.0 was used to assemble and quantify expressed genes and transcripts. PrepDE was used to compute the raw read counts for each gene, and Ballgown v2.6.0 was used to perform multigroup comparisons of gene expression. Rat transcriptomics were computed by

HTSeq v0.6.18, and DESeq2 v1.14.1 was used to identify DEGs between the two groups. In both human PBMC and rat liver transcriptomes, significance was defined as an “adjusted P value < 0.05” in detecting transcript changes, unless indicated otherwise. The FDR calculated with the Benjamini–Hochberg procedure (as implemented in the R function *P*.adjust) was used to control type I error in multiple tests.

Functional enrichment analysis

The annotation enrichment analysis of DEGs was performed using ClueGO in Cytoscape. The Bonferroni step-down method was used to adjust the FDR, and Gene Ontology terms with adjusted P values < 0.05 were considered significantly enriched. To identify candidate genes involved in ACLF pathophysiology, annotation enrichment analysis was carried out on the DEGs, which were differentially expressed in the comparisons of both ACLF versus LC and ACLF versus NC, and the frequencies of genes in the targeted biological processes were counted.

ROC and AUC

The accuracy and sensitivity of prognostic scores are also important to predict outcomes in patients with ACLF. The receiver operating characteristic (ROC) curve is normally used to evaluate the predictive ability (accuracy and sensitivity) of any new prognostic score. This curve plots the sensitivity against 1-specificity (false-positive rate), and the area under the receiver operating characteristic curve (AUROC) is generally stated; the greater the AUC is, the more accurate the test.

Animal experiments

The experimental animals were provided by the Zhejiang Academy of Medical Sciences animal center (animal license number SCXK 2019-0002, Hangzhou, China) and maintained in a specific

pathogen-free facility with controlled environmental conditions (22±1 °C, 55±5% relative humidity, 12 hr. light-dark cycle) with access to standard rodent chow and water ad libitum.

Establishment of conditional THBS1^{KO} mice

To generate hepatocyte-specific THBS1^{KO} mice, the gRNA to the THBS1 gene, the donor vector containing loxP sites, and Cas9 mRNA were co-injected into fertilized mouse eggs to generate targeted conditional knockout offspring. F0 founder animals were identified by PCR followed by sequence analysis, and were bred to WT mice to test germline transmission and F1 animal generation. For breeding and genotyping strategies, the heterozygous targeted mice were crossed to generate homozygous targeted mice, which were bred with tissue-specific Cre-deleted mice, and the offspring mice were screened for THBS1 gene conditional knockout by the same assay described above.

Multicolor immunofluorescence

Tyramide signal amplification (TSA) technology was applied to detect the expression intensities of THBS1, a hepatocyte marker (albumin, ALB) and a hepatic Kupffer cell marker (CD86) using a TSA kit (NEL820001KT, Akoya, Japan) on hepatic tissues by applying the corresponding stains and antibodies using a Leica Bond-Max according to the manufacturer’s instructions (Leica Biosystems Inc., Melbourne, Australia). The generated images were analyzed by SlideViewer software (3DHISTECH Ltd., Hungary).
