# Supplementary material for: Thrombospondin 1 enhances systemic inflammation and disease severity in acute-on-chronic liver failure
Source: BMC Med. 2024 Mar 5;22:95. doi: 10.1186/s12916-024-03318-x (PMC10913480; doi:10.1186/s12916-024-03318-x)

ADDITIONAL FILE 3

Thrombospondin 1 enhances systemic inflammation and disease severity in acute-on-chronic liver failure

Hozeifa Mohamed Hassan†, Xi Liang†, Jiaojiao Xin†, Yingyan Lu, Qun Cai, Dongyan Shi, Keke Ren, Jun Li, Qi Chen, Jiang Li, Peng Li, Beibei Guo, Hui Yang, Jinjin Luo, Heng Yao, Xingping Zhou, Wen Hu, Jing Jiang*, Jun Li*

Contents

- **Supplementary Supplementary original western blots**

Representative western immunoblotting of β-actin in liver tissue collected from WT and THBS1[KO] mice

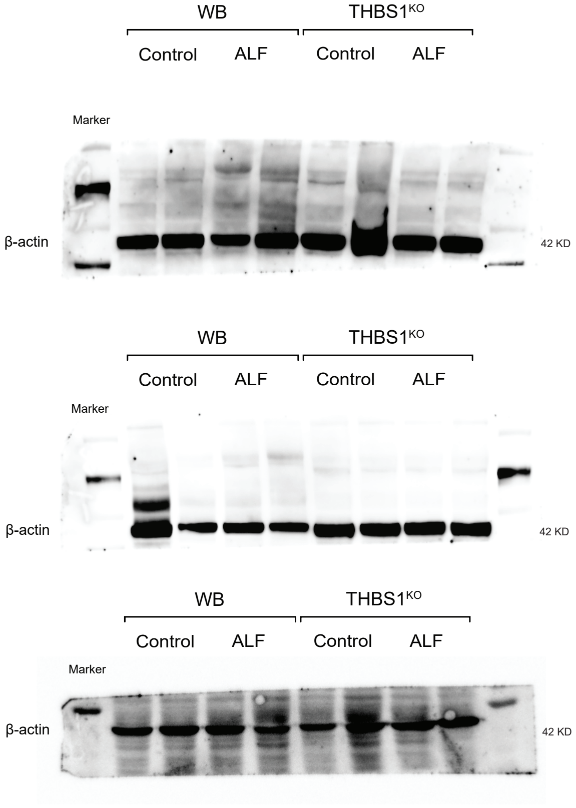

Representative western immunoblotting of THBS1 in liver tissue collected from WT and THBS1^{KO} mice

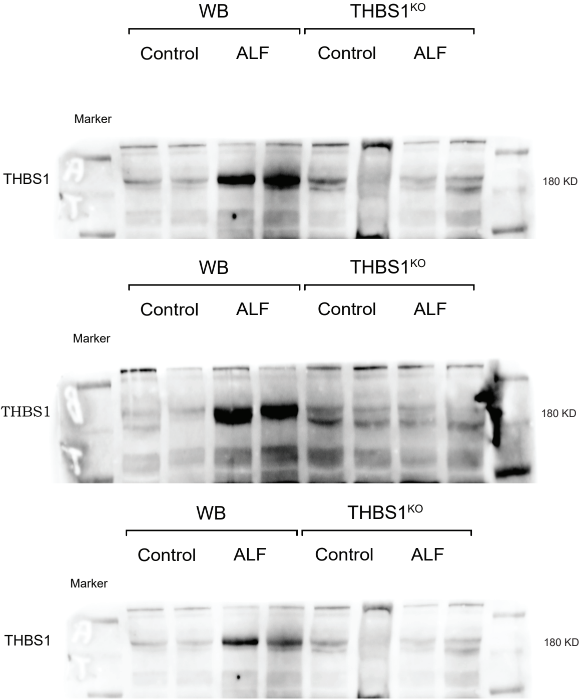

Representative western immunoblotting of cleaved caspase-3 in liver tissue collected from WT and THBS1^{KO} mice

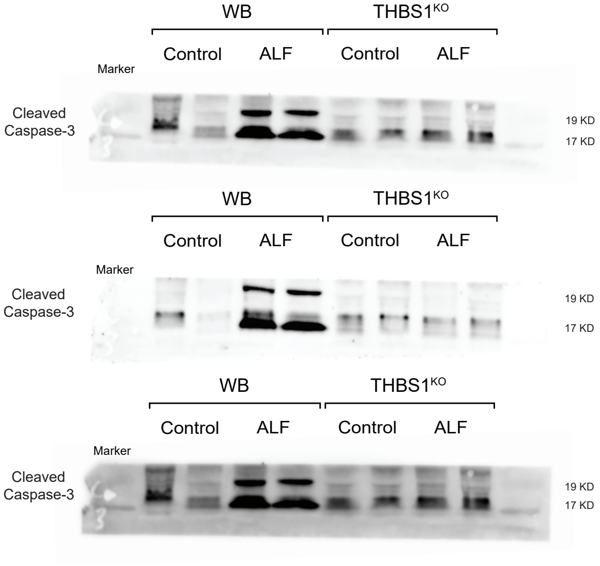

Representative western immunoblotting of BAX in liver tissue collected from WT and THBS1^{KO} mice

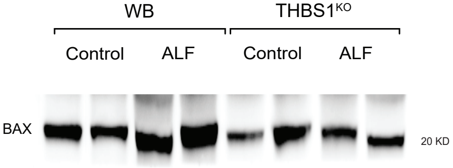

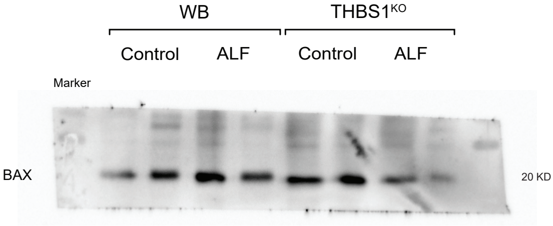

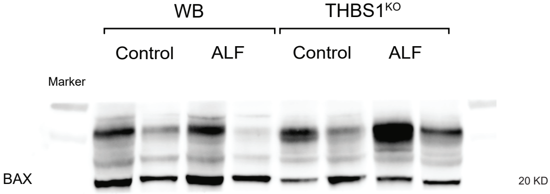

Representative western immunoblotting of Bcl2 in liver tissue collected from WT and THBS1^{KO} mice

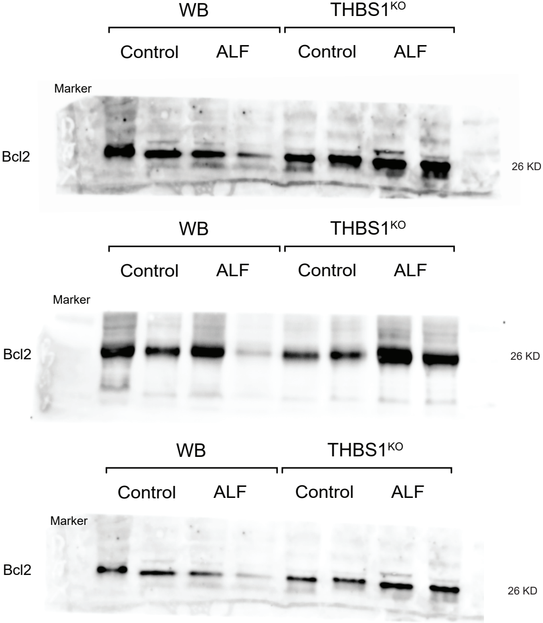

Representative western immunoblotting of β-actin in liver tissue collected from ACLF rats

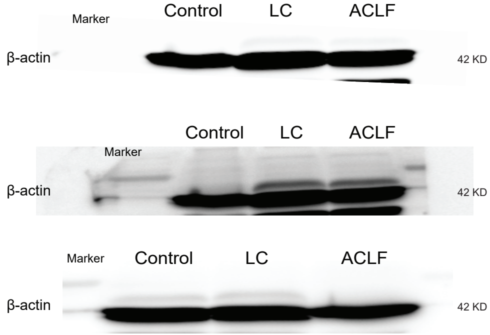

Representative western immunoblotting of THBS1 in liver tissue collected from ACLF rats

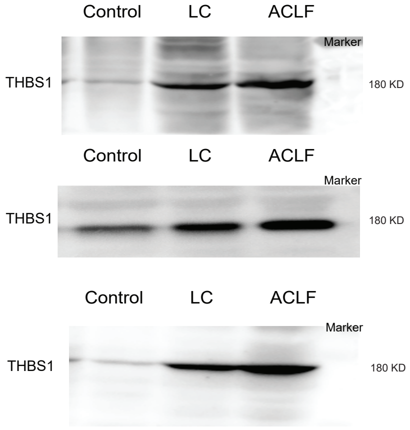

Representative western immunoblotting of BAX in liver tissue collected from ACLF rats

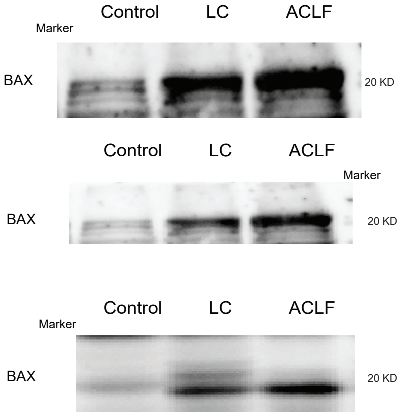

Representative western immunoblotting of cleaved caspase-3 in liver tissue collected from ACLF rats

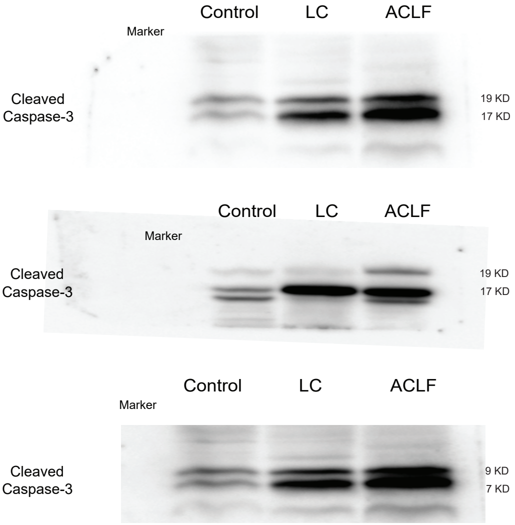

Representative western immunoblotting of Bcl2 in liver tissue collected from ACLF rats

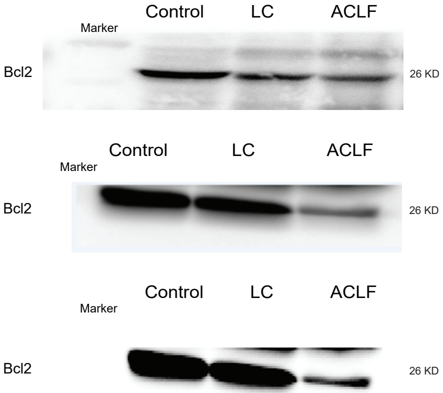

Supplement: Supplementary file 3 — Additional file 3: Data File. Supplementary original western blots. [file 12916_2024_3318_MOESM3_ESM.pdf]
