# Supplementary material for: Thrombospondin 1 enhances systemic inflammation and disease severity in acute-on-chronic liver failure
Source: BMC Med. 2024 Mar 5;22:95. doi: 10.1186/s12916-024-03318-x (PMC10913480; doi:10.1186/s12916-024-03318-x)
Supplement: Supplementary file 4 — Additional file 4: Fig S1. Correlations between THBS1 gene with inflammatory and apoptotic markers in the human PBMC transcriptome. [file 12916_2024_3318_MOESM4_ESM.pdf]

SUPPLEMENTARY FILE 4

Thrombospondin 1 enhances systemic inflammation and disease severity in acute-on-chronic liver failure

Hozeifa Mohamed Hassan†, Xi Liang†, Jiaojiao Xin†, Yingyan Lu, Qun Cai, Dongyan Shi, Keke Ren, Jun Li, Qi Chen, Jiang Li, Peng Li, Beibei Guo, Hui Yang, Jinjin Luo, Heng Yao, Xingping Zhou, Wen Hu, Jing Jiang*, Jun Li*

Contents

- **Figure S1**

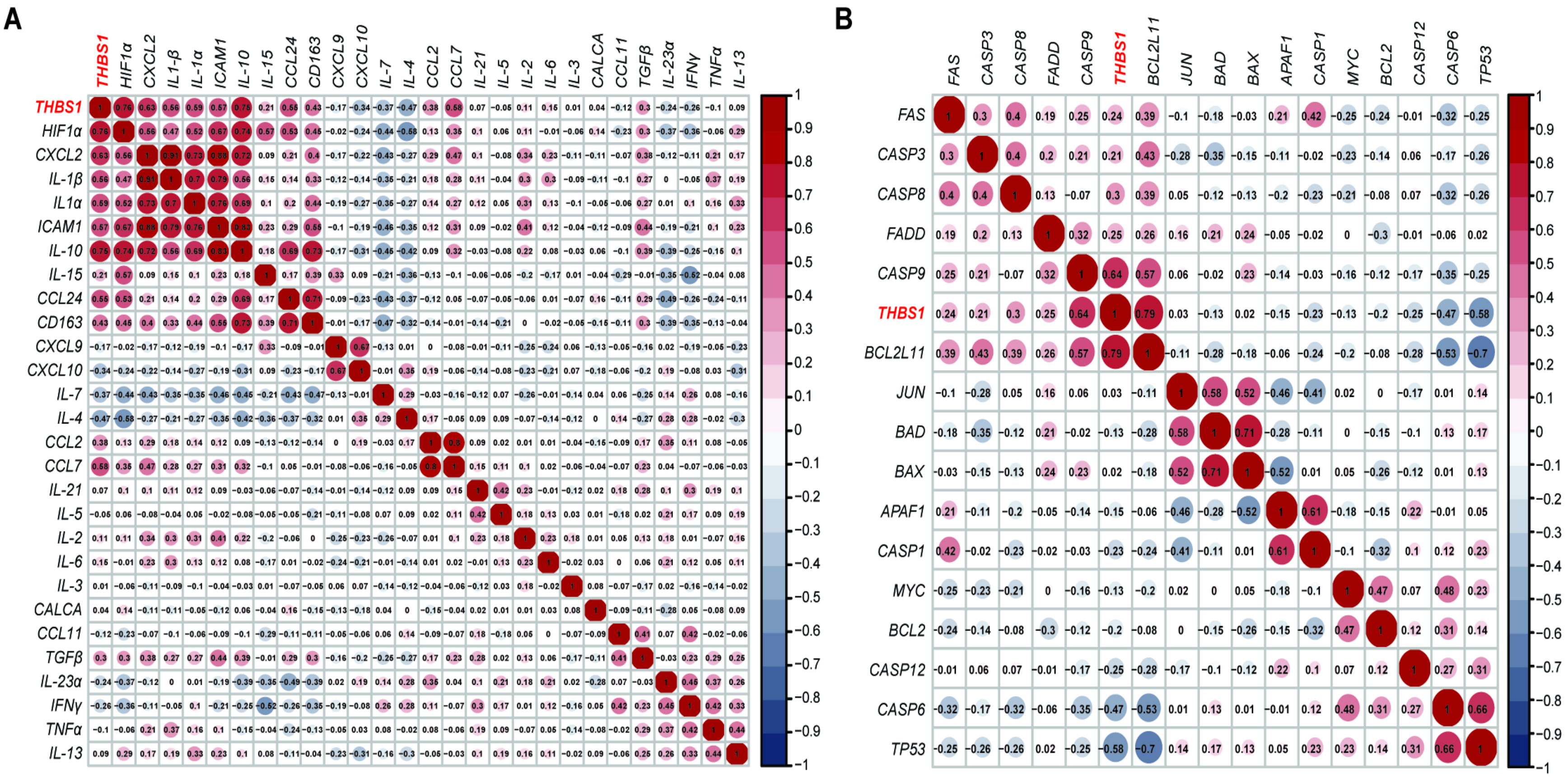

Fig.S1 Correlations between THBS1 with inflammatory and apoptotic markers. THBS1 was positively correlated with other top-key genes related to (**A**) Immune micro-environment and (**B**) Apoptotic markers in the human PBMCs transcriptome.
